# Complex Multistate Photophysics of a Rhodanine Photoswitch

**DOI:** 10.1002/anie.202506137

**Published:** 2025-07-31

**Authors:** Anam Fatima, Pratip Chakraborty, Xinyue Xu, Garth A. Jones, Isabelle Chambrier, Giorgia Logan, Andrew N. Cammidge, Trevor Smith, Christopher R. Hall, Stephen R. Meech

**Affiliations:** ^1^ School of Chemistry University of East Anglia Norwich Norfolk England; ^2^ Department of Chemistry University of Melbourne Melbourne Victoria Australia

**Keywords:** Excited state dynamics, Photoswitch, Quantum chemical, Triplet, Ultrafast

## Abstract

Development of new and improved photoswitches for molecular photonics and photo‐pharmaceutics is an increasingly important research objective. Recently a promising family of photoswitches based on the rhodanine motif was described. Here, the photophysics of a typical example are investigated by ultrafast UV and IR spectroscopy and quantum chemical calculations. Remarkably, the photophysics are very different to and more complex than those of closely related monomethine photoswitches, which relax by ultrafast internal conversion to the electronic ground state. In the rhodanine photoswitch, the allowed Franck–Condon excited state also relaxes on a sub‐picosecond timescale, but the ground state is repopulated only after several hundred picoseconds. Instead, the Franck–Condon state relaxes through (at least) two intermediate states. These states are characterized by transient spectroscopy, and the reaction pathway is modeled by quantum chemical calculations. Comparison of calculated and measured IR data suggests that a triplet mediated isomerization pathway is responsible for the slow excited state dynamics. The triplet state is rapidly populated via coupling of a nearly degenerate nπ* state populated by ultrafast internal conversion from the bright ππ* state. This unexpected isomerization pathway has important implications for the synthesis, analysis, and application of rhodanine photoswitches.

## Graphical abstract

Slow switching. A novel photoswitch is observed by ultrafast experiment to have unusually slow excited state dynamics. The pathway is resolved by quantum chemical calculation.

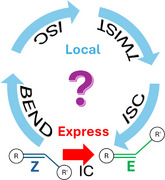

## Introduction

Photoswitches are key components in the development of molecular photonics. They have diverse applications in light‐controlled phenomena, ranging over catalysis,^[^ ^1^, ^2^ ^]^ chirality,^[^ ^3^ ^]^ photopharmacology,^[^ ^4^, ^5^, ^6^ ^]^ photoactive gels,^[^ ^7^, ^8^ ^]^ superesolution bioimaging,^[^ ^9^, ^10^ ^]^ and nanomanipulation.^[^ ^11^ ^]^ There is significant synthetic effort being devoted to enhancing the performance of photoswitches, typically targeting improved yields, higher photostability, and the development of switching ability in the visible region of the spectrum.^[^ ^12^, ^13^, ^14^, ^15^, ^16^, ^17^, ^18^ ^]^ The latter is especially important in photopharmacology and bioimaging applications, given the deleterious effects of UV radiation on living systems and its poor penetration depth.

**Figure 1:**
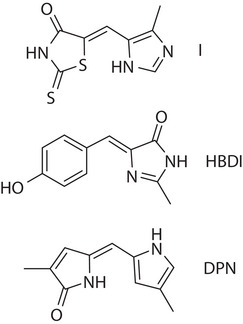
The rhodanine‐based photoswitch **(I)** studied in this work, shown with two well‐studied monomethine‐based photoswitches: HBDI (the green fluorescent protein chromophore) and DPN (a bilin chromophore).

Recently, Köttner et al. described a new family of photoswitches based around the rhodanine motif; an example is shown in Figure 1.^[^ ^19^ ^]^ These new photoswitches have good photostability and operate efficiently under blue light irradiation, having absorption spectra with maxima in the 380–400 nm region and large transition dipole moments. Further, it was shown that these rhodanine photoswitches are characterized by high yields of Z→E and E→Z photoisomerization with long (at least hours) thermal recovery times. In a specific application, the photoswitch was shown to support light activated apoptosis.^[^ ^19^ ^]^ Here, we resolve the excited state dynamics underlying the operation of a representative rhodanine photoswitch through ultrafast electronic and vibrational spectroscopy and electronic structure calculations. The objective is to provide mechanistic information for the future synthesis and exploitation of enhanced examples. The sample chosen for study (Figure 1) was synthesized following the method of Köttner et al. and is labelled **I** (it was called Z‐8 in the original publication).^[^ ^19^ ^]^ This example was selected for detailed study because of its redshifted absorption, high yield of isomerization, and the well‐separated absorption spectra of its two isomers.

The rhodanine photoswitches are a part of the much larger family of monomethine dyes.^[^ ^20^, ^21^ ^]^ In Figure 1, two other well studied examples are shown, the chromophore of the green fluorescent protein (HBDI) and dipyrrinone (DPN).^[^ ^22^, ^23^, ^24^, ^25^, ^26^, ^27^, ^28^, ^29^ ^]^ Both dyes have been the subject of detailed experimental and theoretical characterization and were shown to undergo Z–E photoisomerization. Indeed, HBDI was the inspiration for the development of other novel monomethine photoswitches,^[^ ^30^ ^]^ while DPN is a key element of the bilin chromophore,^[^ ^31^ ^]^ which is an important switch in photobiology. Both HBDI and DPN exhibit extremely weak fluorescence, which was shown to arise from rapid radiationless decay of the excited states by internal conversion (IC) back to the electronic ground state potential energy surface, on which the product isomer may be stabilized.^[^ ^22^, ^23^, ^24^, ^25^, ^26^, ^27^, ^28^, ^29^ ^]^ Further, neither HBDI nor DPN exhibit viscosity dependent emission or significant solvatochromism. On the basis of its structural similarity and observed weak fluorescence and medium independence, we expected that **I** would show similar excited state dynamics to these other monomethine dyes. The results described below in fact reveal a richer, more complex photoisomerization pathway for **I**.

Of course, the monomethine family of dyes is very broad and its members exhibit different photophysical properties dependending on the nature of the substituent.^[^ ^32^ ^]^ An obvious structural analogue of **I** are the monomethine thioindigoid dyes, which may indeed have been one of the inspirations for the synthesis of the rhodanine photoswitches.^[^ ^19^, ^33^ ^]^ Thioindigiod dye photophysics have been rather thoroughly studied, and they were shown to exhibit moderate fluorescence yields and to have lifetimes of tens of picoseconds, which are sensitive to solvent viscosity and a strong function of polarity.^[^ ^34^, ^35^, ^36^ ^]^ Thus, despite the structural analogy, the thioindigoids are a less suitable model for **I** than HBDI and DPN. However, we show below that **I** also has very different photophysics to the thioindigoids, highlighting its unique behavior.

## Results and Discussion

**Figure 2:**
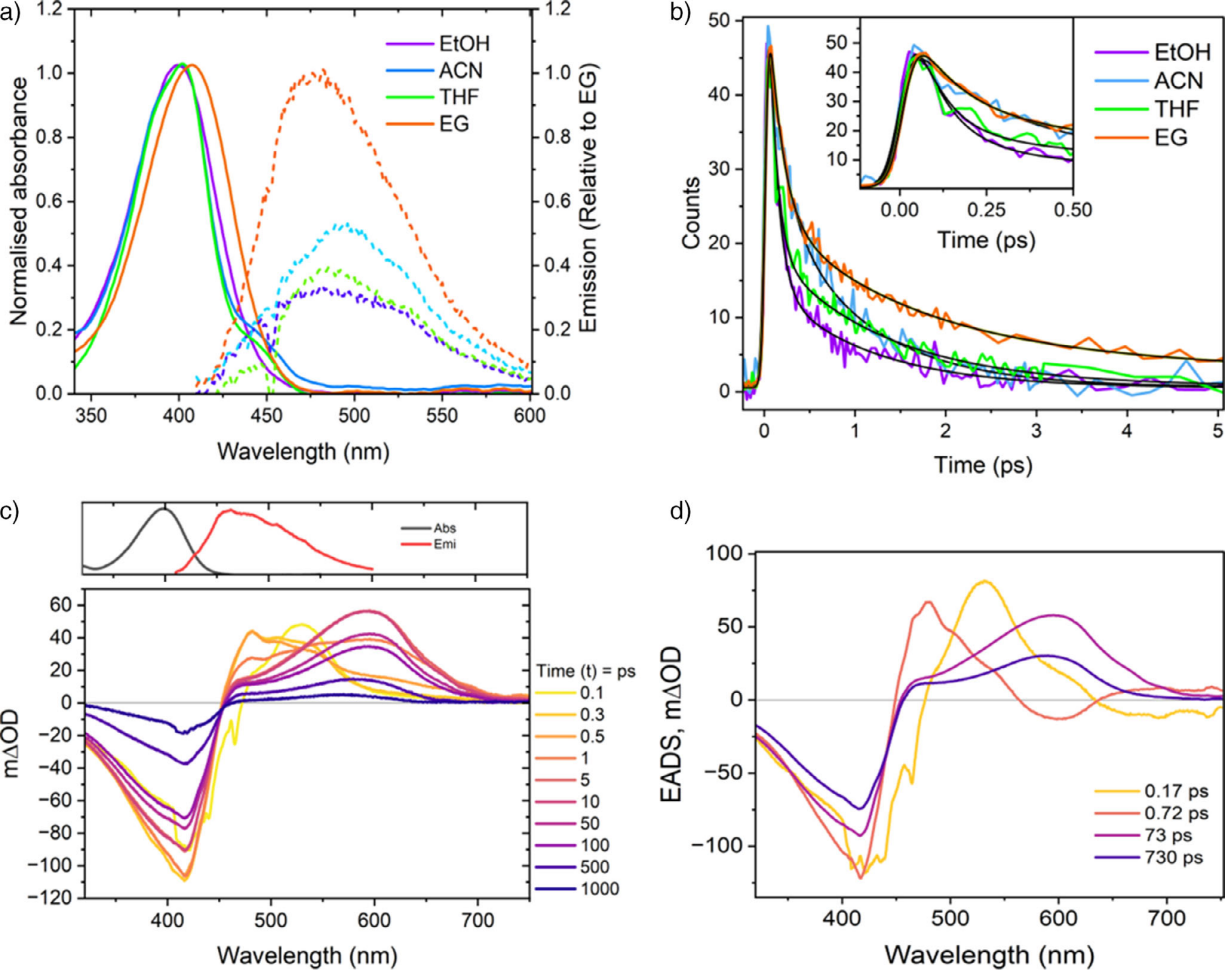
a) Steady‐state absorption and emission spectra (*λ*_exc_ = 400 nm) of **I** in different solvents at room temperature. Emission spectra are normalized to ethylene glycol (EG) to indicate the relative fluorescene yield. b) Ultrafast fluorescence upconversion of **I** excited at 400 nm in different solvents, with time‐resolved emission recorded at the peak wavelength of each emission spectrum. Inset shows a magnified view of the sub‐ps decay. c) Transient absorption spectra of **I** in methanol at different time delays after the pump pulse, with steady‐state absorption and emission spectra included for comparison with ground‐state bleaching (GSB) and stimulated emission (SE). The slight structure in the 0.1 ps spectrum is a result of the coherent artifact. d) Evolution‐associated difference spectra (EADS) derived from global fitting of the transient absorption data.

Figure 2 shows the steady state and time resolved electronic spectroscopy of **I**. The absorption and emission spectra (Figure 2a) are rather featureless. The absorption is strong (extinction coefficient at 401 nm reported as 37800 M^−1^cm^−1^ in THF^[^ ^19^ ^]^ and slightly solvent dependent), while the emission is extremely weak, with an estimated quantum yield of <10^−4^, but somewhat enhanced in viscous media. (The “structure” in emission above 400 nm arises from an imperfect subtraction of solvent Raman.) The fluorescence decay was measured with sub 50 fs resolution by fluorescence up‐conversion to resolve the earliest part of the excited state decay, i.e., the relaxation of the bright (i.e., strongly allowed) Franck–Condon state (Figure 2b). The fluorescence decay is ultrafast and nonsingle exponential in all solvents studied. Qualitatively, the excited state decay is only weakly solvent dependent, although somewhat lengthened in viscous ethylene glycol (EG) consistent with the steady‐state emission (Figure 2a). Fitting the data to a minimum number of exponential terms reveals a dominant (weight > 60%) sub 200 fs component along with a weaker second component of ca. 1 ps (the data for the other solvents are tabulated in Table S1). In EG, a third longer component is detected, but even in that case the mean lifetime is only 5‐fold longer than in ethanol even though the viscosity has increased by a factor of 15. These data suggest a moderate to weak viscosity dependence for the excited state decay with no obvious large polarity dependence. Such an ultrafast fluorescence decay with a weak solvent polarity and viscosity dependence is typical of some other monomethine photoswitches, including HBDI and DPN.^[^ ^25^, ^29^ ^]^

In contrast, transient absorption (TA) data reveal a significant divergence between the photophysics of **I** and those of other monomethine dyes (Figure 2c). In these TA difference spectra, a positive signal indicates formation of a new intermediate or product state while a negative (or bleach) signal indicates a species removed from the ground state or a stimulated emission (SE). The TA spectra for **I** in methanol show that ground state recovery (GSR) is incomplete even after 1 ns (Figure 2c). This contrasts sharply with TA of other monomethine dyes, where the dominant decay pathway is ultrafast IC to the ground state in ca 0.1–5 ps, followed by vibrational cooling on the time scale of a few ps. The TA of **I** show that evolution occurs on the excited state surface for several hundred ps after the sub‐ps bright state decay before the eventual ground state repopulation in >1 ns. The initial TA shows a bleach of the ground state at 410 nm accompanied by an excited state absorption (ESA) at 530 nm. The ESA appears within the 100 fs time resolution of the experiment so can be assigned to the Franck–Condon excited state. This initial state relaxes in <1 ps to a new intermediate with a broad ESA peaking near 480 nm. That intermediate relaxes in a few picoseconds to populate a state with a broad ESA centred at 600 nm, which finally relaxes back to the electronic ground state in hundreds of ps, undergoing a small blue shift (ca 30 nm) with accompanying refilling of the GSB (Figure 2c). The data for solvents acetonitrile, THF and EG are qualitatively very similar with only the first intermediate being less well resolved in EG (Supporting Information (SI) Figure S1, Table S2).

The TA data were globally analyzed in terms of a sequence of first order steps and the resulting evolution associated difference spectra (EADS) for **I** in methanol are shown in Figure 2d with the quality of the global fit shown in SI (Figure S2). Analyses for the other solvents are also shown in the SI (Figure S3). In methanol, there is an initial ultrafast (170 fs) relaxation associated with a blue shift in the TA, which is assigned to ultrafast decay from the bright Franck–Condon state. This blueshifted EADS relaxes in 720 fs to a redshifted EADS. We associate these two‐time constants with the very similar values in the time resolved fluorescence (Figure 2b, Table 1). Since the ground state is not recovering in parallel with these processes, the evolution must occur in the excited state. Thus, the 170 fs decay reflects a decay in the S_1_‐S_0_ transition dipole moment to form a weakly emissive “dark” state. The 1 ps decay of this “dark” state leads to a new redshifted intermediate, which itself relaxes in 73 ps to a final intermediate, from which the ground state recovers with a 730 ps time constant. The 73 ps relaxation to the final state is associated with only small changes in EADS, specifically a blue shift, and it is possible this indicates a nonsingle exponential decay of a distribution of intermediate structures formed in ca 1 ps from the “dark” state.

**Table 1:** Lifetimes of **I** obtained from different techniques in methanol.

	τ_1_ (ps)	τ_2_ (ps)	τ_3_ (ps)	τ_4_ (ps)	τ_5_ (ps)
Fluo Upconv	0.09	1.10	–	–	–
TA	0.17	0.72	73	730	–
TRIR	–	0.90	24	684	Inf

The data and EADS in acetonitrile, THF and ethylene glycol are very similar to those in methanol (Figure S3), although there is a less clear distinction of the earliest EADS. Quantitatively (Table S2) the first two‐time constants recovered are in very good agreement with the time resolved fluorescence data (Table S1) and independent of solvent viscosity and polarity. The two longer decay times are somewhat viscosity dependent, which may indicate that those processes involved large scale structure change, consistent with an isomerization reaction, as discussed below.

**Figure 3:**
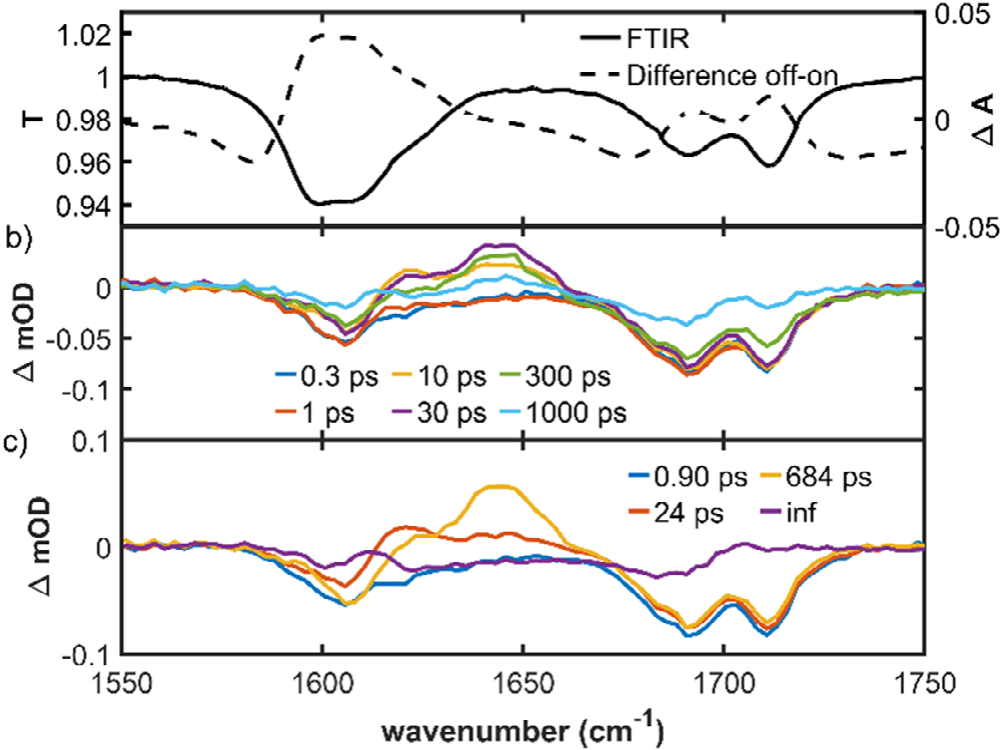
a) Steady‐state IR transmission of **I** in CD_3_OD compared to the light minus dark difference spectrum. b) TRIR at selected times after excitation at 400 nm. c) EADS recovered from a sequential analysis of the data in (b). The time constants associated with each step are shown, where inf implies product with a lifetime much greater than the measurement range product.

To better characterize these long‐lived intermediates in the relaxation pathway of **I**, we measured transient infra‐red (TRIR) difference spectra in deuterated methanol (CD_3_OD) with 250 fs resolution. The steady‐state IR spectra are shown in Figure 3a, and the time resolved data in Figure 3b.

The initial (300 fs) TRIR of **I** is dominated by the ground state bleach (GSB), specifically a pair of IR active modes around 1700 cm^−1^ and another broad bleach at 1600 cm^−1^, which align well with the steady‐state IR spectrum (Figure 3a). Within 10 ps, a pair of transient absorption signals have developed at 1620 and 1645 cm^−1^. With increasing delay time, the 1620 cm^−1^ transient decays and the 1645 cm^−1^ one grows, before finally relaxing to refill the GSB. As the ground state refills, a new much longer‐lived final TA develops at 1615 cm^−1^. The data were fit to the sequential global analysis model used for the TA but including a final spectrum to account for the long‐lived product observed at 1615 cm^−1^. The EADS recovered (Figure 3c, Table 1) show an initial state dominated by the three GSB modes, relaxing in 0.9 ps to yield a transient signal at 1620 cm^−1^, which forms a second intermediate in 24 ps in which the transient absorption has shifted to 1645 cm^−1^, bisecting the two ground state bleach features at 1600 and 1700 cm^−1^. These relaxation steps occur without any filling of the grounds state bleach modes. This state goes on to form the final product in 684 ps accompanied by almost complete refilling of the ground state. We compare the steady‐state light minus dark IR difference spectrum with the final (product plus residual bleach) EADS in the Supporting Information (Figure S4). The overall shapes are similar, but the profiles are slightly different, and the EADS bands are blueshifted by ca 10 cm^−1^, which suggests that there are even slower components in the relaxation, occurring on time scales longer than those measured here.

The TRIR data confirm that photoswitch **I** indeed operates by relaxing through a series of long lived (compared to typical monomethine dyes) excited state intermediates. The 0.9 ps relaxation and the 684 ps long‐lived intermediate are in good agreement with the TA data (Table 1) although the fastest (ca 0.2 ps) component seen in fluorescence and TA is not resolved because of the lower time resolution, so the 0.9 ps step in TRIR reflects the decay of the rapidly formed “dark” excited state. The agreement for the formation time of the long‐lived intermediate is less good (24 ps against 73 ps in TA), but this may reflect either the accuracy attainable with the current TRIR data (the fit quality at representative wavenumbers is shown in Figure S5) or that the true kinetics are more complicated, involving a nonsingle exponential distribution of intermediate states, which are only approximately represented by this single step. The tens of ps evolution seen (Figure 3b,c) in the TRIR shows that the intermediate state(s) have distinct vibrational spectra even though the TA (the third and fourth EADS) are similar (Figure 2c).

To summarize, the excited state isomerization of **I** involves relaxation from the bright state to a “dark” excited state intermediate in ca 200 fs, resolved as *τ*_1_ in fluorescence and TA. The “dark” state so formed undergoes further relaxation in ca 1 ps (*τ*_2_) to form a second intermediate that undergoes relaxation in the excited state in tens of picoseconds (*τ*_3_) to form a long‐lived intermediate, which ultimately decays to the ground state (either the original Z isomer or the E product) in several hundred picoseconds (*τ*_4_); the time constants associated with each measurement are collected in Table 1. There are two plausible explanations for these unexpectedly slow and complex kinetics. One is the presence of a lower lying optically forbidden singlet state below the bright state, which is populated by S_2_–S_1_ IC in ca 1 ps and decays in tens to hundreds of picoseconds to the ground/product state. Such behavior has been seen in for example diphenyl polyenes, where the allowed S_2_ excited state decays in <1 ps to a dark (symmetry forbidden) ππ* state with a nanosecond lifetime.^[^ ^37^, ^38^ ^]^ In the case of **I**, which is of low symmetry but is heteroaromatic, a more likely assignment for the initial low‐lying dark state would be a ^1^ nπ* state. The second possibility is that the isomerization is triplet state mediated, and the bright state decay is an intersystem crossing (ISC) to the triplet manifold, on which the structural evolution occurs, ultimately repopulating the ground and product states by reverse ISC. This requires a ca 1 ps ISC, which is certainly exceptionally fast for ISC in the absence of a heavy atom, but by no means unprecedented, for example, in the case of nearly degenerate singlet and triplet nπ*/ππ* states.^[^ ^39^, ^40^ ^]^ Indeed, in the monomethine thioindigoids, a minor population of the triplet states has been observed even in the absence of nπ* states, although these molecules have much longer excited state lifetimes than **I** and the triplet state is very long‐lived (unreactive).^[^ ^41^ ^]^

**Figure 4:**
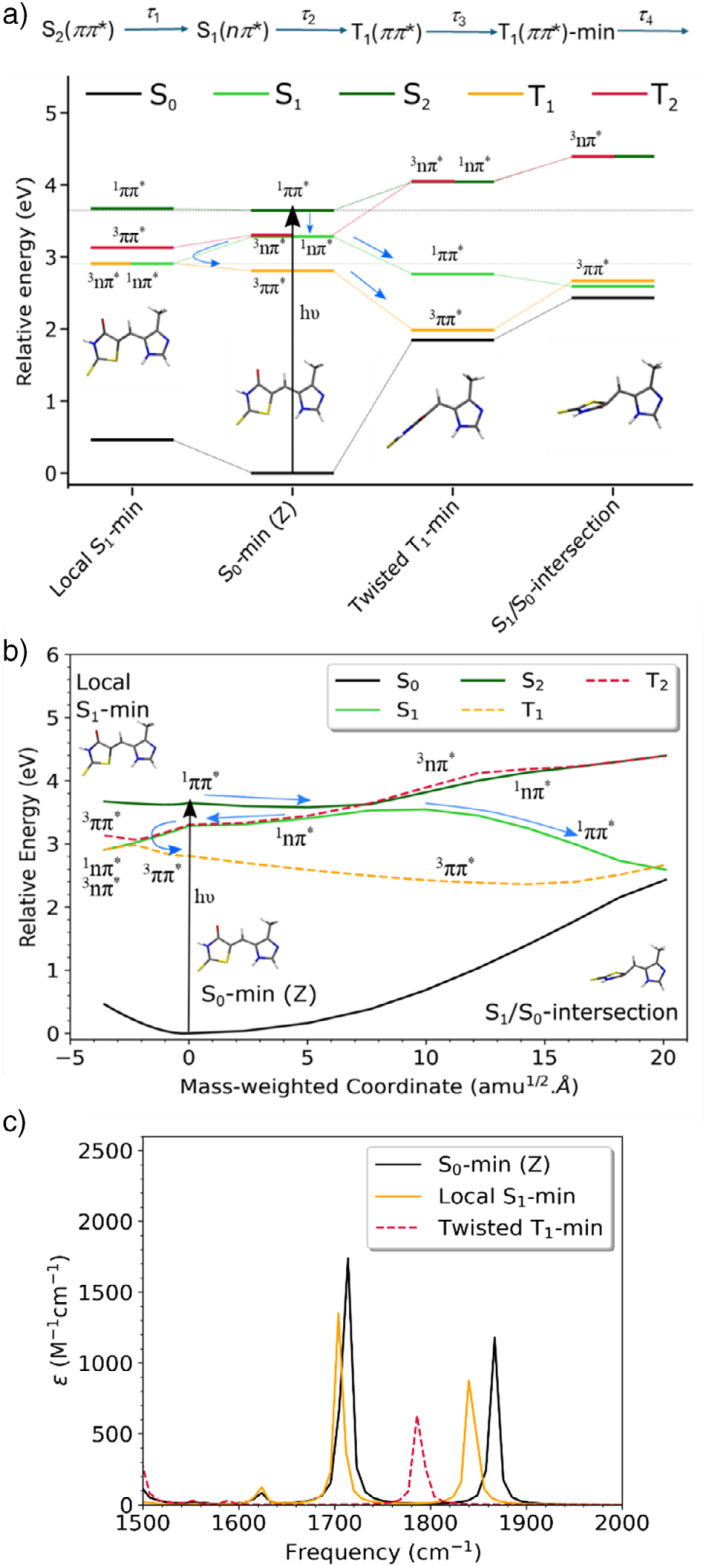
a) Relative‐energies (with respect to S_0_‐min (Z)) of critical points calculated at XMS‐CASPT2/SA(5/3)‐CAS(14,12)/cc‐pVDZ level of theory along with the character of the states (nπ* or ππ*). The scheme hypothesized for the relaxation dynamics is also shown (top) with the experimental time‐constants indicated. b) Relative energies (with respect to S_0_‐min (Z)) of the linearly interpolated pathways from S_0_‐min (Z) to local S_1_‐min and S_1_/S_0_‐intersection region, calculated at the XMS‐CASPT2/SA(5/3)‐CAS(14,12)/cc‐pVDZ level of theory along with the character of the states (nπ* or ππ*), SA(5/3) refers to averaging over five‐singlet and three‐triplet states. Singlet states and triplet states are illustrated using solid and dashed lines respectively. This diagram illustrates that after photoexcitation to S_2_ and internal conversion to S_1_, there are two main pathways for the population on S_1_ to end up on T_1_ state and c) IR spectra between 1500–2000 cm^−1^ for the critical points calculated at ωB97x‐D functional and 6–31G(d,p) basis set.

To resolve these relaxation pathways, we turn to quantum chemical calculations, and those data are summarized in Figure 4. All the critical points of the photo‐switch on the ground, excited singlet and triplet states were optimized at the density functional theory (DFT) level using ωB97x‐D functional and 6–31G(d,p) basis set,^[^ ^42^, ^43^, ^44^, ^45^ ^]^ whereas the local minimum on the S_1_ state was optimized at the time‐dependent TD‐DFT level using the same functional^[^ ^46^, ^47^, ^48^ ^]^ and basis set. Frequency calculations were performed at the same levels of theory to ensure the stationary points obtained are the minima. S_1_/S_0_ conical intersection optimization was attempted at the complete active space self‐consisted field (CASSCF)^[^ ^49^ ^]^ level using cc‐pVDZ^[^ ^50^ ^]^ basis set and an active space of 12 electrons in 10 orbitals. Vertical excitation energies (VEE) and oscillator strengths were calculated at all the critical points at the high‐level extended multistate complete active space second‐order perturbation (XMS‐CASPT2)^[^ ^51^, ^52^, ^53^ ^]^ theory using an active space of 14 electrons in 12 orbitals and cc‐pVDZ basis set for a comparison of the excited state energies. For a more detailed description, refer to Supporting Information. The (TD)‐DFT calculations were carried out in Gaussian16,^[^ ^54^ ^]^ while the high‐level multireference calculations were carried out using BAGEL^[^ ^55^ ^]^ and OpenMolcas.^[^ ^56^, ^57^ ^]^ The frequency calculations yield the IR spectra for comparison with Figure 3.

For the S_0_ ground state, two prominent high wavenumber modes are calculated in the IR spectrum at 1711 and 1866 cm^−1^, which correlates well with the IR spectrum in acetonitrile‐d_3_ (Figure S7), although the calculated wavenumbers are uniformly higher than experimentally observed by ∼100 cm^−1^. These two vibrations arise from the C═O (1866 cm^−1^) and C═C (1711 cm^−1^) stretch modes (Figure S6). The highest wavenumber C═O stretch in methanol is observed to split into two bands in the steady‐state IR spectrum, which is also reflected in the GSB TRIR data (Figure 3a,b). This result suggests a specific solvent interaction but modeling it would require calculations with explicit solvent molecules at several H‐bonding sites, making the calculations excessively difficult.

Consistent with the mechanism described above, the calculation shows a ^1^ nπ* S_1_ state lying energetically 2900 cm^−1^ below a bright ^1^ ππ* S_2_ state. Thus, the fastest, sub 200 fs, decay observed in fluorescence and TA (Figure 2b, *τ*_1_ in Table 1) can be assigned to fast S_2_ (bright) to S_1_ (“dark”) state IC. The S_1_ state is calculated to be degenerate with a ^3^ nπ* state and close to a ^3^ ππ* (Figure 4a). However, the S_1_ state populated by IC is itself unstable and can relax along two possible barrierless energetically downhill pathways. One leads to an S_1_/S_0_ conical intersection (CoIn) as found in typical monomethine dyes. This pathway would thus lead to ultrafast IC, which is observed experimentally in HBDI and DPN but not in **I** (Figures 2 and 3). This is most likely because in **I** this pathway is associated with a large structural reorganization. The alternative low volume structure change pathway involves out of plane motion of C═S and reaches a stable point on the excited state surface, which remains close to ^3^ nπ* and ^3^ ππ* states (Figure 4a,b). The IR spectrum, at this state, local S_1_‐min, was calculated and has the same two high frequency stretch modes at wavenumbers very similar to the ground state, only slightly red shifted by 5–15 cm^−1^ (Figure 4c). This small shift is consistent with the experimental observation (Figure 3b,c) that the earliest EADS of the inhomogeneoulsy broadened TRIR spectrum resembles the ground state bleach. We can further conclude that this local S_1_‐min state is not the long‐lived state, which has a very different IR spectrum (Figure 3b). However, from this stable point, calculations show that the relaxed excited state can evolve further through ISC to populate a ^3^ ππ* state, which provides the lowest energy pathway from local S_1_ minimum (Figure 4a,b). This fast ISC must occur on the 0.7–1 ps time scale (*τ*_2_) resolved in the slower fluorescence decay component and in the TA and TRIR (Figures 2 and 3 and Table 1). We ascribe this fast ISC to the nearly degenerate nπ* and ππ* states of different character (Figure 4a).^[^ ^58^ ^]^ Thus, the first pathway, to the S_1_/S_0_ CoIn, which would lead to rapid IC, is inconsistent with TA and TRIR of **I**. Instead, the observed slow excited state dynamics of **I** favors the second low volume structure change pathway, remaining on the excited state surface to rapidly populate a triplet state from S_1_‐min in ca 1 ps (*τ*_2_). That the low volume structure change pathway supports fast ISC is also shown by the spin‐orbit coupling matrix elements (SOCME) calculations illustrated in Figure S8 and Tables S3 and S4, which show the increasing strength of SOCME between S_1_ and T_1_ along the low volume structure change making this pathway more probable for ISC (unlike the decreasing SOCME along the high volume structure change pathway).

However, the pathway is complicated with multiple changes in the character of the states (see Figure 4b), so quantum dynamical calculations are required to more rigorously resolve the initial dynamics and branching ratios; such calculations are planned.

The ^3^ ππ* state populated from the local S_1_‐min (Figures 4a and S7) is calculated to be unstable with respect to twisting about the methine C═C bond. Thus, the triplet state will relax along the isomerization coordinate (among others) in several tens of picoseconds (*τ*_3_) on the relatively flat T_1_ surface. This relaxation time may reflect solvent friction experienced by the large‐scale structure change consistent with the dependence on solvent H‐bonding and solvent viscosity (Table S2), although the calculation suggests a barrierless pathway. An almost half twisted (97°) minimum (Twisted T_1_‐min) is detected, and its IR spectrum was calculated (Figure 4c). The spectra again show the C═O and C═C stretch modes, but now both are markedly redshifted such that the wavenumber for the carbonyl stretch lies between those for the two S_0_‐min modes (Figure 4b). This is in good agreement with the experimental TRIR difference spectrum and the EADS for the long‐lived transient (Figure 3b,c). The C═C stretch is calculated to have shifted below 1500 cm^−1^ taking it out of the experimental TRIR window (Figure 3b). Thus, the energetics, the relatively slow excited state dynamics and the agreement between calculated and observed TRIR all point to a triplet mediated pathway for the isomerization of **I**. The twisted T_1_‐min is reached from local S_1_‐min by large scale intramolecular structural reorganization displacing solvent (*τ*_3_). Of course, the triplet twisted state must still undergo reverse ISC to form the E product isomer (or refill the Z ground state). This pathway is not calculated, but the TA suggests the ground state is largely repopulated within a few ns, requiring moderately fast reverse ISC; this relaxation (*τ*_4_) was measured as ca 700 ps. To probe, this further requires calculations that explicitly model ISC, but we note that Twisted T_1_‐min on the triplet surface lies close to the twisted singlet ground state (Figure 4a) energy, which may serve to enhance ISC. Finally, we note that analysis of the molecular orbitals indicates that the lone pair on the S atom is involved in the ^1^ nπ* state (Figure S8). Thus, it will be interesting to see whether the oxorhodanines (which lack the C═S bond) exhibit different excited state dynamics.^[^ ^19^ ^]^ The required synthesis and measurements are in progress. Indeed, all of the other monmethine dyes described lack this C═S source of nonbonding electrons.

Finally, we turn to consideration of the E isomer, the other half of the photoswitch. A photostationary state of E–Z isomers populated by 365 nm irradiation was studied by excitation on the red‐edge to favor excitation of the E isomer. The less than perfect separation of the electronic spectra of E and Z forms in alcohols (compared to THF^[^ ^19^ ^]^) and the broad bandwidth inevitably associated with sub 100 fs pulses makes confirmation of selective excitation challenging. However, the TRIR spectra, the EADS, and their associated time constants recovered from global analysis are very similar to those for the Z‐isomer (see Figure S12) and confirm that a similar mechanism operates in the E form, including population of the unexpected long‐lived intermediate. The corresponding energetics were calculated as for Figure 4b and are shown in Figure S13. Consistent with the TRIR spectra, they show a similar level structure to the Z form, notably with regards to singlet—triplet degeneracy and barrier along the singlet pathway. Dynamics calculations will provide a more quantitative picture. On the basis of these data, we suggest that the E and Z forms have a common triplet mediated isomerization mechanism.

## Conclusion

The excited state dynamics of a recently developed rhodanine photoswitch have been analyzed by ultrafast electronic and vibrational spectroscopy and quantum chemical calculations. The dynamics are unusually slow compared to photoisomerization in previously studied monomethine dyes. Both experiments and calculations point to an intersystem crossing mediated isomerization pathway, in contrast to the fast IC, which typically dominates in similar monomethine dyes. This has implications for the design of similar systems and the possible side reactions that may play a role in applications of these photoswitches. For example, this relatively long‐lived triplet state may undergo reactions that would not occur in other monomethine dyes. Secondly, the role of ^1^ nπ* state may lead to an unexpected substituent dependence, for example, revealing heavy atom effects or dependence of photophysics on singlet‐triplet energy gap. The calculations point to an important role for nonbonding electrons of the C═S group in modifying the photophysics of **I**, so it may be possible to introduce other sources of nonbonded electrons.

## Conflict of Interests

There are no conflicts of interest to declare.

## Supporting information



Supporting Information

## Data Availability

The data that support the findings of this study are available from the corresponding author upon reasonable request.
