# Supplementary material for: Complex Multistate Photophysics of a Rhodanine Photoswitch
Source: Angew Chem Int Ed Engl. 2025 Jul 31;64(37):e202506137. doi: 10.1002/anie.202506137 (PMC12416450; doi:10.1002/anie.202506137)
Supplement: Supplementary file 1 — Supporting Information [file ANIE-64-e202506137-s001.docx]

**Complex Multistate Photophysics of a Rhodanine Photoswitch**

Anam Fatima^1^, Pratip Chakraborty^1^, Xinyue Xu^2^, Garth A. Jones^1^, Isabelle Chambrier^1^, Giorgia Logan^1^, Andrew N. Cammidge^1^, Trevor Smith^2^ Christopher R. Hall^2^, and Stephen R. Meech*^1^

^1^School of Chemistry University of East Anglia

^2^Department of Chemistry University of Melbourne

*Author for correspondence: [s.meech@uea.ac.uk](mailto:s.meech@uea.ac.uk)

Contents

[1. Experimental section 3](#_Toc198835793)

[a.) Steady-State Absorption and Emission 3](#_Toc198835794)

[b.) Fluorescence up-conversion 3](#_Toc198835795)

[c.) Visible transient absorption (TA) 3](#_Toc198835796)

[d.) Time-resolved infra-red (TRIR) 4](#_Toc198835797)

[e.) Quantum Chemical Calculations 4](#_Toc198835798)

[f.) Rhodanine photoswitch NMR....................................................................................................5](#_Toc198835798)

[2. Data Analysis: Figures and Tables 6](#_Toc198835799)

[Table S1: Time-constants (*τ*) and amplitudes (*α*) obtained by fitting the TRF decay traces using a sum of exponential function. <τ> denotes the average time of decay. 6](#_Toc198835800)

[Fig. S1 Transient absorption spectra of **I** in (a) acetonitrile and (b) ethylene glycol at different time delays after the pump pulse, with steady-state absorption and emission spectra included for comparison of ground-state bleaching (GSB) and stimulated emission (SE). 7](#_Toc198835801)

[Fig. S2 Global exponential fit (four sum of exponential model) of kinetic traces presented at selected wavelengths in methanol. 8](#_Toc198835802)

[Fig. S3 Evolution-associated difference spectra (EADS) derived from global fitting of the kinetic traces of the transient absorption data in (a) acetonitrile, (b) THF, and (c) ethylene glycol; (d), (e) and (f) show corresponding global exponential fit of kinetic traces presented at selected wavelengths in the respective solvents. 9](#_Toc198835803)

[Table S2. Comparison of the lifetimes of I, obtained from transient absorption spectroscopy, across different solvents as a function of their dielectric constant (*ε*) and viscosity (*η*). 10](#_Toc198835804)

[Fig. S4. The Final EADS from the data analysis (Figure 3) is compared with the light minus dark difference spectrum recorded in FTIT. The same bands are observed but there is clearly some additional relaxation between 3 ns and the final spectrum. 11](#_Toc198835805)

[Fig. S5. Time resolved IR data at key wavenumbers are compared with the fit (black lines) recovered form the global analysis. 11](#_Toc198835806)

[Fig. S6 Experimental FTIR recorded in deuterated MeCN 12](#_Toc198835807)

[Fig. S7 Displacement vectors illustrating normal modes associated with C=C stretch (calculated at 1711 cm^-1^) and C=O stretch (1866 cm^-1^) for S_0_-min (Z). 12](#_Toc198835808)

[Fig. S8 Relative energies (with respect to ground state energy at S0-min (Z)) along linearly interpolated pathway from S0-min (Z) to S1/S0-intersection region and to the local S1-min. 13](#_Toc198835809)

[Fig. S9: Dominant orbital transitions along with their weights (in parenthesis) associated with S_1_ (nπ*) and S_2_ (ππ*) states 14](#_Toc198835810)

Fig. S10. Active space orbitals used in the XMS-CASPT2/CAS(14,12)/cc-pVDZ single point calculations.......................................................................................................................................15

[Fig S11: Important critical points along key geometrical parameters and atom-indexing used. Pyramidalization at C_13_ is required to reach the local S_1_-min, whereas pyramidalization at C_10_ is required alongside twist to reach the vicinity of S_1_/S_0_-intersection. There is also a reversal of the bridging C-C bonds from singlet to triplet minima. Only four (S_0_-min (Z), local S_1_-min, twisted T_1_-min, S_1_/S_0_-intersection) out of the six located critical points have been used to explain the possible pathways after photoexcitation. 16](#_Toc198835811)

[Figure S12. Transient IR data for and E + Z photostationary state formed in CD_3_OD after irradiation at 365 nm. The pump wavelength was 350 nm. The data are strikingly similar to those in Figure 3 (Z state) suggesting similar kinetics for the E excited state relaxation. 1](#_Toc198835812)7

Fig S13. The calculations from Fig 4 and S8 extended to include the E isomer.................................17

Table S3. SOCME (cm^-1^) for important singlet-triplet transitions along the LIIC between S_0_-min (Z) and local S_1_-min. Maximum of the S_1_-T_1_ SOCME is highlighted.**....................................................17**

# Experimental section

## Steady-State Absorption and Emission

The UV–Visible ground-state absorption spectra were measured using a PerkinElmer Lambda XLS spectrophotometer with 1 cm path length quartz cuvettes, ensuring absorbance remained below 0.1 (<2.5 μM). Emission spectra at room temperature were recorded on an Edinburgh Instruments FS5 spectrofluorometer in a right-angle configuration, with a 5 nm bandwidth for both excitation and emission. Emission was weak and the contribution from solvent Raman was subtracted.

## Fluorescence up-conversion

The ultrafast fluorescence upconversion experiment, described in detail elsewhere^[1]^, employs a Coherent Micra-10 femtosecond oscillator, a mode-locked Ti:Sapphire laser operating at ~800 nm with a pulse duration of ~20 fs, a repetition rate of 76 MHz, and an output power of ~750 mW. Sample excitation was at 400 nm achieved via second-harmonic generation, with an average power of up to 9 mW at the sample. The upconversion process (800 nm + fluorescence) occurs in a 100-µm BBO crystal, yielding a temporal resolution of <50 fs, as determined from the upconversion of solvent Raman scattering.

## Visible transient absorption (TA)

The transient absorption setup used here has been detailed elsewhere^[2]^. The pump and probe beams were generated using an 800 nm fundamental output beam produced by a Spectra Physics-Mai Tai Laser oscillator and amplified by a Ti:Sapphire regenerative amplifier (Spectra Physics-Spitfire ACE). This amplified output pulse, characterized by a duration of 100 fs at 800 nm, a repetition rate of 1 kHz, and an energy of 5 mJ per pulse, served to drive the optical parametric amplifier (OPA, Light Conversion TOPAS Prime), thereby producing a tuneable pump pulse for sample excitation. A white light continuum (WLC) probe was generated by focusing part of the fundamental 800 nm beam onto a 3 mm thick CaF_2_ window, yielding a broadband continuum ranging from 380 to 750 nm. The window was mounted on a two axis translation stage to prevent optical damage. Transient absorption spectra presented in this work were recorded following sample excitation at 410 nm. The energy of the pump beam at the sample cell was attenuated to 300 μW (300 nJ/pulse). All measurements were conducted in 1 mm cuvettes with OD <1 (<concentration 25 μM). Samples were either static or flowed and no difference was observed.

## Time-resolved infra-red (TRIR)

For femtosecond transient infrared (TRIR) spectroscopy measurements, the pump and probe were generated via non-linear optical processes using femtosecond duration pulses at 1030 nm from a Ytterbium-based laser (Pharos, Light Conversion) operating at 10kHz. An optical parametric amplifier (OPA, Orpheus, Light Conversion) with harmonic generator (Lyra, Light Conversion) generate pump pulses with extremely tuneable wavelengths in the UV-NIR region (210-2660 nm, ~150 fs temporal FWHM, ~5 nm bandwidth). A mechanical chopper (Thorlabs, MC1F60) was used to modulate the pump pulses at 5 kHz. The pump beam passed through a mechanical delay stage (Newport ESP300) to obtain optical delay times from -50 to 1000 ps, then focused to achieve ~350 μm FWHM spot at sample position. Linear polarisers were in place to set the relative polarisation between the pump and the probe at magic angle. The mid-IR probe (4000-13000 nm, <180 fs temporal FWHM, fundamental repetition rate 100 kHz) was generated by an OPA and DFG (ORPHEUS TWINS, Light Conversion). The entire probe beamline between the laser system and MIR detector was purged with dry nitrogen to reduce the influence of unwanted atmospheric MIR absorption bands. TRIR spectra were obtained with an imaging spectrometer (Horiba iHR320) and liquid nitrogen cooled mercury-cadmium-telleride (MCT) detector (FPAS 128-element MCT detector, Infra-Red Systems). The measurements were conducted in a flow cell (Harrick Cell) with a 10 mL reservoir (2.5 mM, OD ~ 0.9 at 400 nm, optical path 150 μm). To obtain reasonable signal to noise ratio, the pump fluence was adjusted to 420 μJ/cm^2^ or 200 nJ/pulse.

## Quantum Chemical Calculations

Most of the critical points (S_0_-min (Z), S_0_-min (E), Twisted T_1_-min, Planar T_1_-min) of the photo-switch on the ground singlet and triplet state were optimized at the density functional theory (DFT)^[3],[4]^ level using ωB97x-D^[5]^ functional and 6-31G(d,p)^[6]^ basis set, whereas the local minimum on the S_1_ state (Local S_1_-min) was optimized at the time-dependent TD-DFT^[7–9]^ level using the same functional and basis set. Frequency calculations were performed at the same levels of theory to ensure the obtained stationary points are the minima. These optimizations were performed using Gaussian16 software^[10]^ and the critical point geometries with relevant geometrical parameters are illustrated in Fig. S11. S_1_/S_0_ conical intersection optimization was attempted at the complete active space self-consisted field (CASSCF)^[11]^ level using cc-pVDZ^[12]^ basis set and an active space of 12 electrons in 10 orbitals (CAS(12,10)) with a five-state averaging using BAGEL^[13],[14]^ package. Although it did not converge, it reached S_1_-S_0_ energy difference of 0.002 eV at the SA5-CASSCF(12,10)/cc-pVDZ level of theory to provide us with an understanding of the geometrical distortions associated with the intersection (S_1_/S_0_-intersection). Substantial pyramidalization of C_10_ is also associated with the S_1_/S_0_-intersection in addition to the twist motion. Vertical excitation energies (VEE) and oscillator strengths were calculated at all the critical points at the high-level extended multistate complete active space second-order perturbation (XMS-CASPT2) theory^[15–17]^using OpenMolcas^[18,19]^. Five singlet states and three triplet states were averaged using an active space of 14 electrons in 12 orbitals (Fig. S10) and cc-pVDZ basis set for a comparison of the excited state energies and characters at the critical points. IPEA shift was set to 0 a.u., whilst the imaginary shift was set to 0.3 a.u. to remove intruder states. Linear interpolations were carried out in internal coordinates (LIIC) between the S_0_-min (Z) and the local S_1_-min, and between the S_0_-min (Z) and the S_1_/S_0_-intersection. Fig. S8 illustrates the linearly interpolated pathways along those two directions at the above mentioned XMS-CASPT2/SA(5/3)-CAS(14,12)/cc-pVDZ level. Spin-orbit coupling matrix elements (SOCME) were also calculated using the XMS-CASPT2 wavefunctions along the LIIC, employing OpenMolcas. The linearly interpolated path was further extended from the S_1_/S_0_-intersection to S_0_-min (E) and further towards the local minimum on S_1_ near S_0_-min of the E isomer (Local S_1_-min (E)) using the same level of theory as before (Fig. S13).

1. Rhodanine photoswitch I

Synthesised following the reported route (ref 8 from original MS, Kottner JACS 2024 1894). ^1^H NMR (Acetone-d6, 500 MHz) 11.62 (br s, 1H), 7.78 (s, 1H), 7.49 (s, 1H), 2.84 (br s, 1H), 2.49 (s, 3H) ppm. ^13^C NMR (Acetone-d6, 100.62 MHz) 200.8, 169.71, 137.39, 134.99, 133.87, 123.41, 122.01, 9.57 ppm.


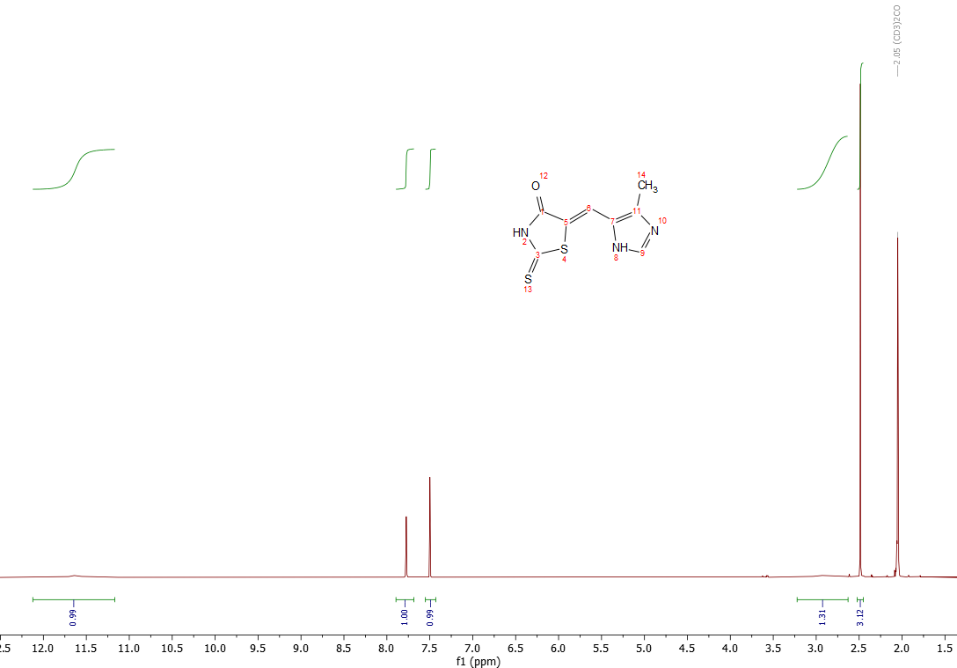


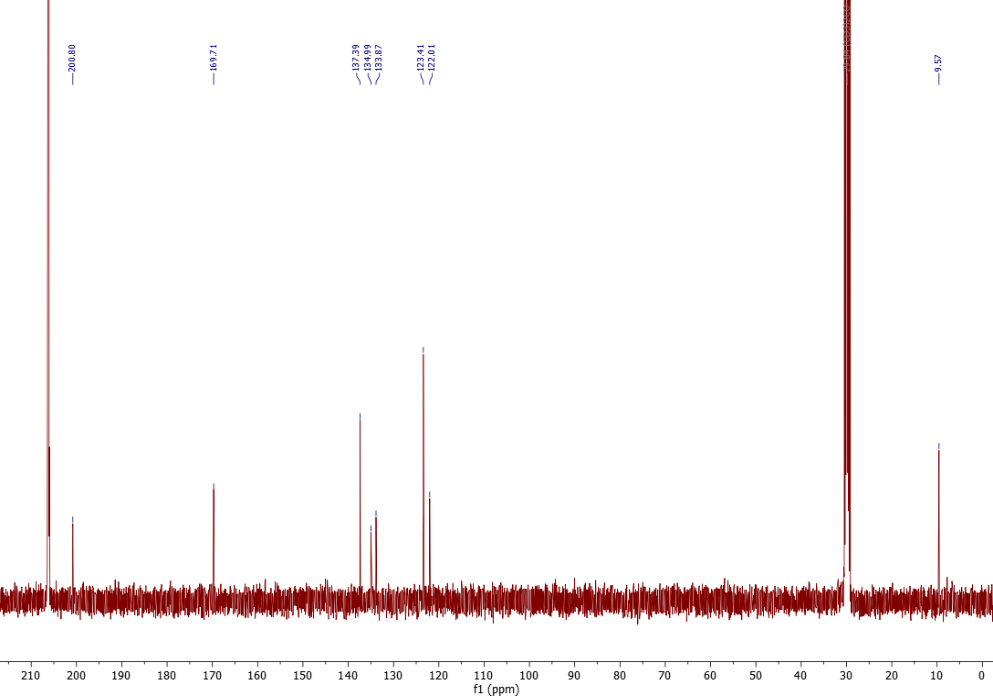


# Data Analysis: Figures and Tables

## Table S1: Time-constants (*τ*) and amplitudes (*α*) obtained by fitting the TRF decay traces using a sum of exponential function. <τ> denotes the average time of decay.

| **Solvents** | **α_1_** | **τ_1_ /ps** | **α_2_** | **τ_2_ /ps** | **α_3_** | **τ_3_ /ps** | **<τ> /ps** |
| --- | --- | --- | --- | --- | --- | --- | --- |
| **EtOH** | 60 | 0.09 | 14 | 1.1 | -- | -- | 0.3 |
| **ACN** | 32 | 0.18 | 29 | 0.9 | -- | -- | 0.5 |
| **THF** | 54 | 0.08 | 19 | 1.2 | -- | -- | 0.4 |
| **EthGly** | 82 | 0.15 | 40 | 1.5 | 11 | 10 | 1.4 |


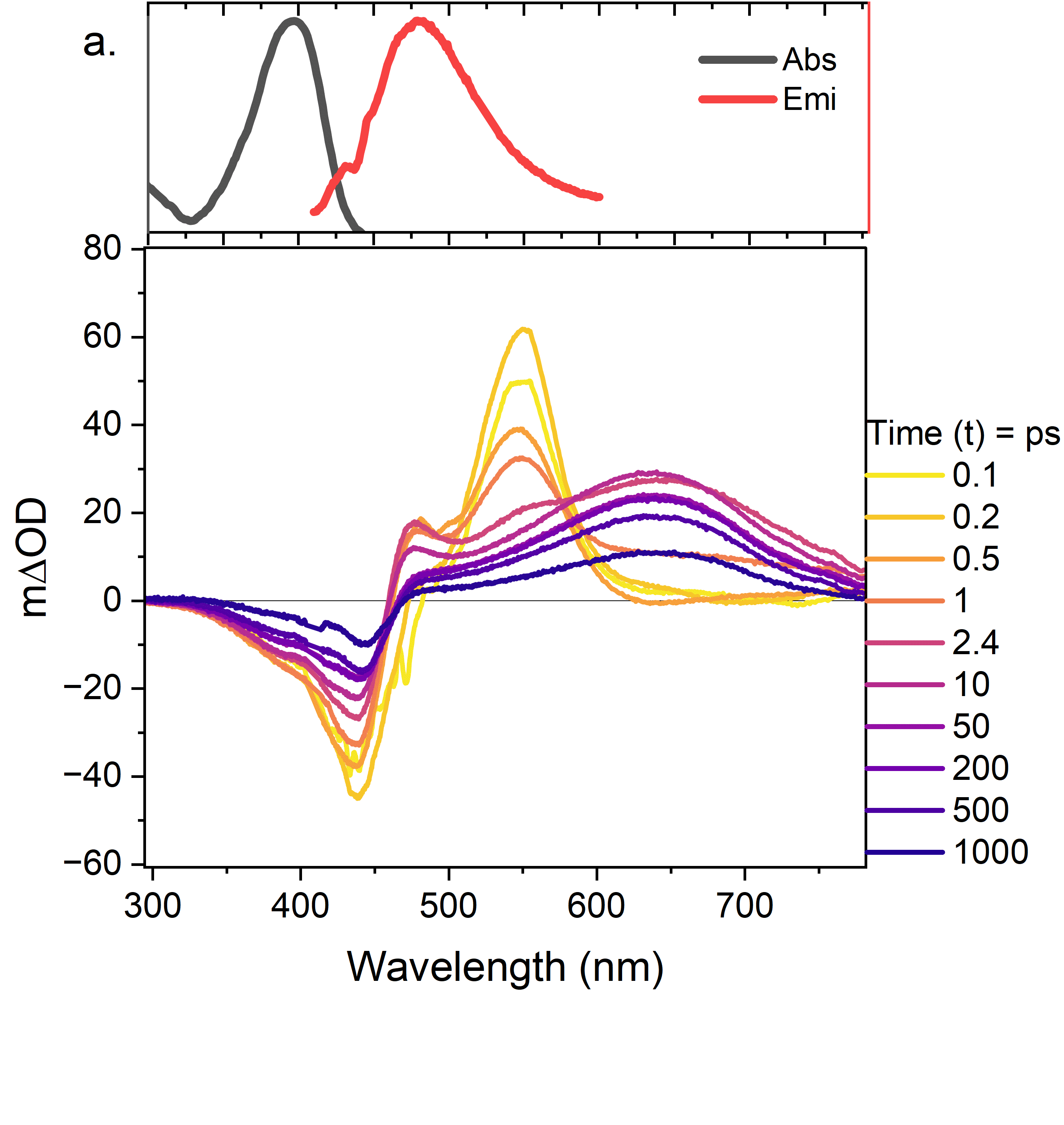

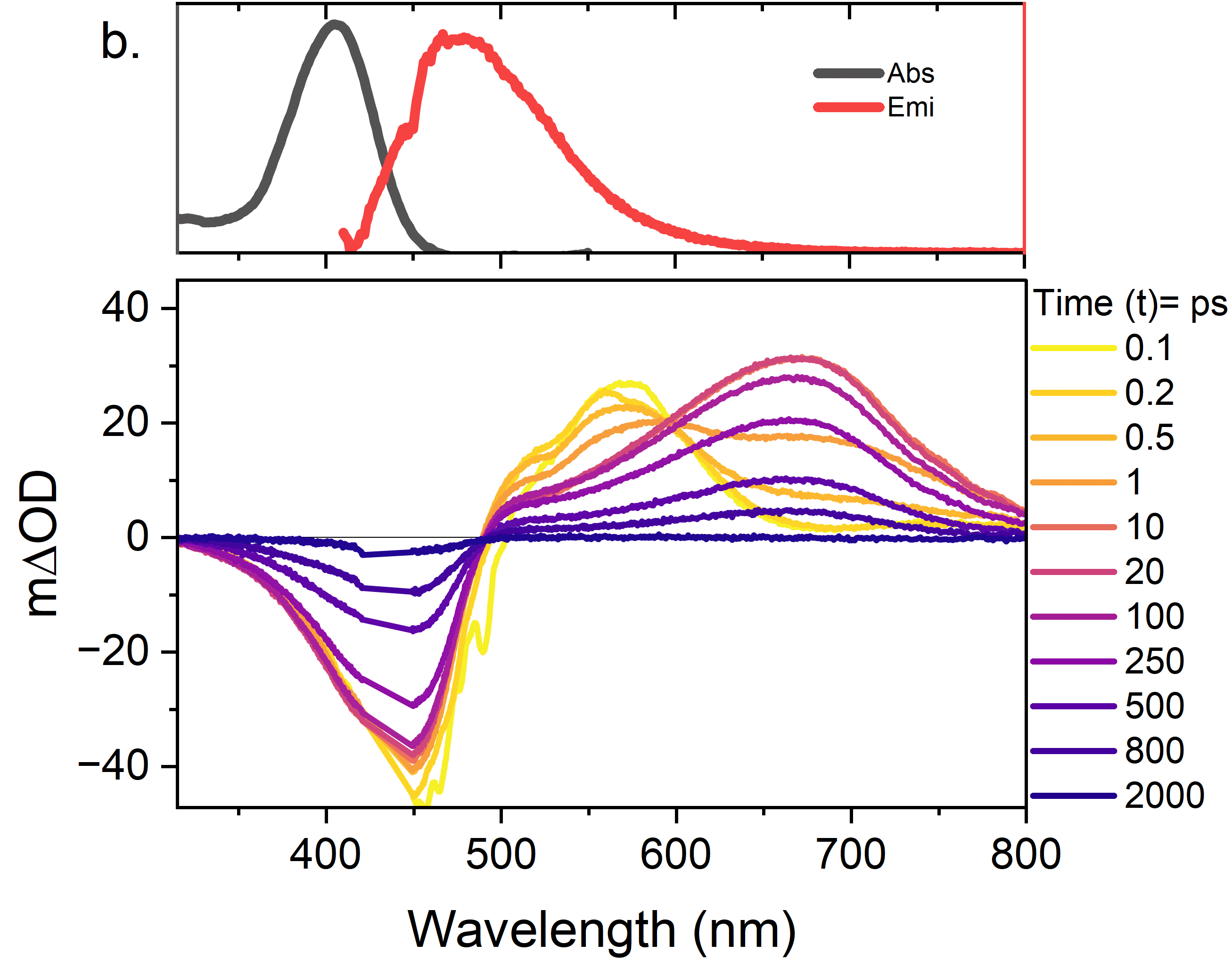


## Fig. S1 Transient absorption spectra of **I** in (a) acetonitrile and (b) ethylene glycol at different time delays after the pump pulse, with steady-state absorption and emission spectra included for comparison of ground-state bleaching (GSB) and stimulated emission (SE).

## Fig. S2 Global exponential fit (four sum of exponential model) of kinetic traces presented at selected wavelengths in methanol.


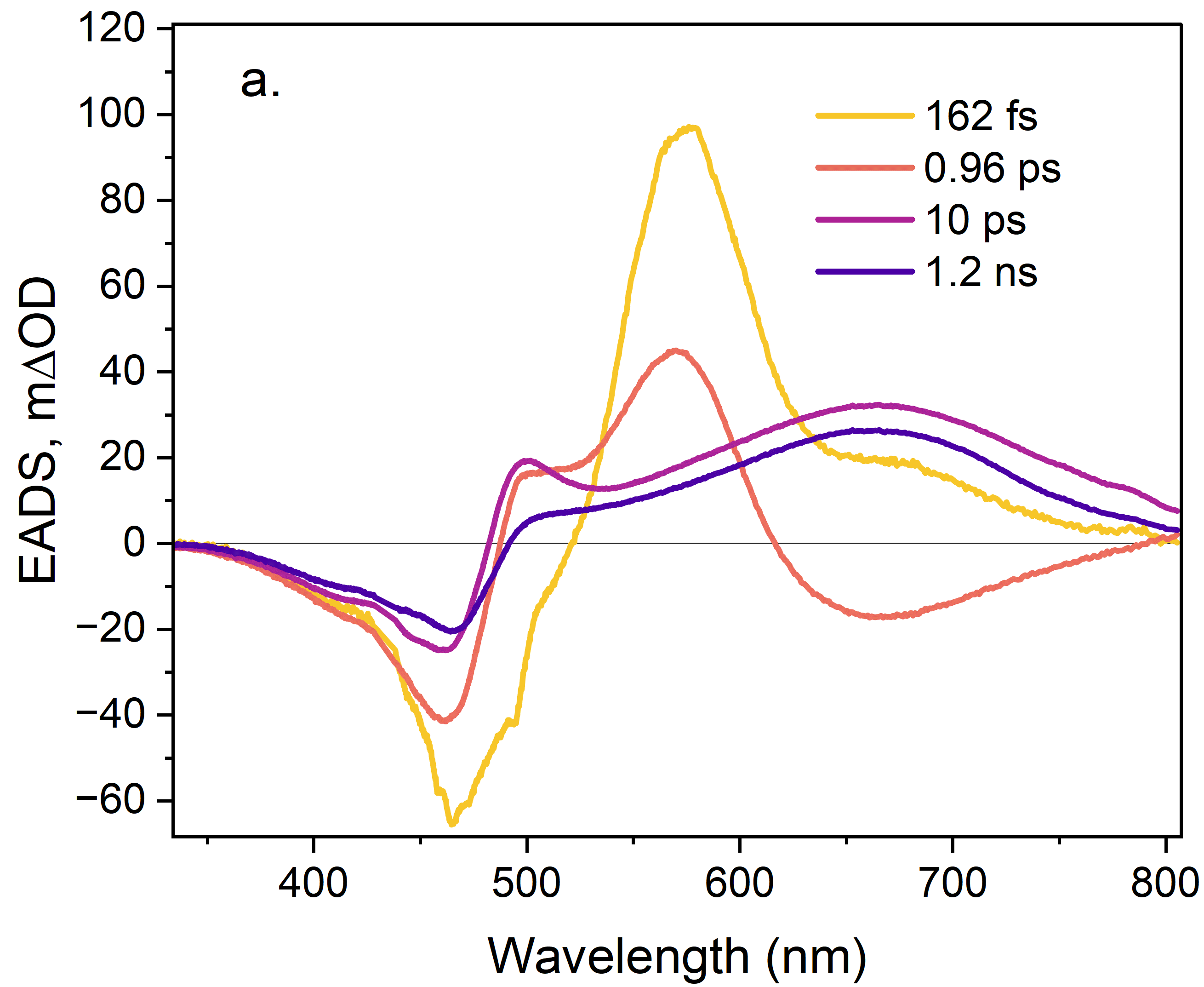

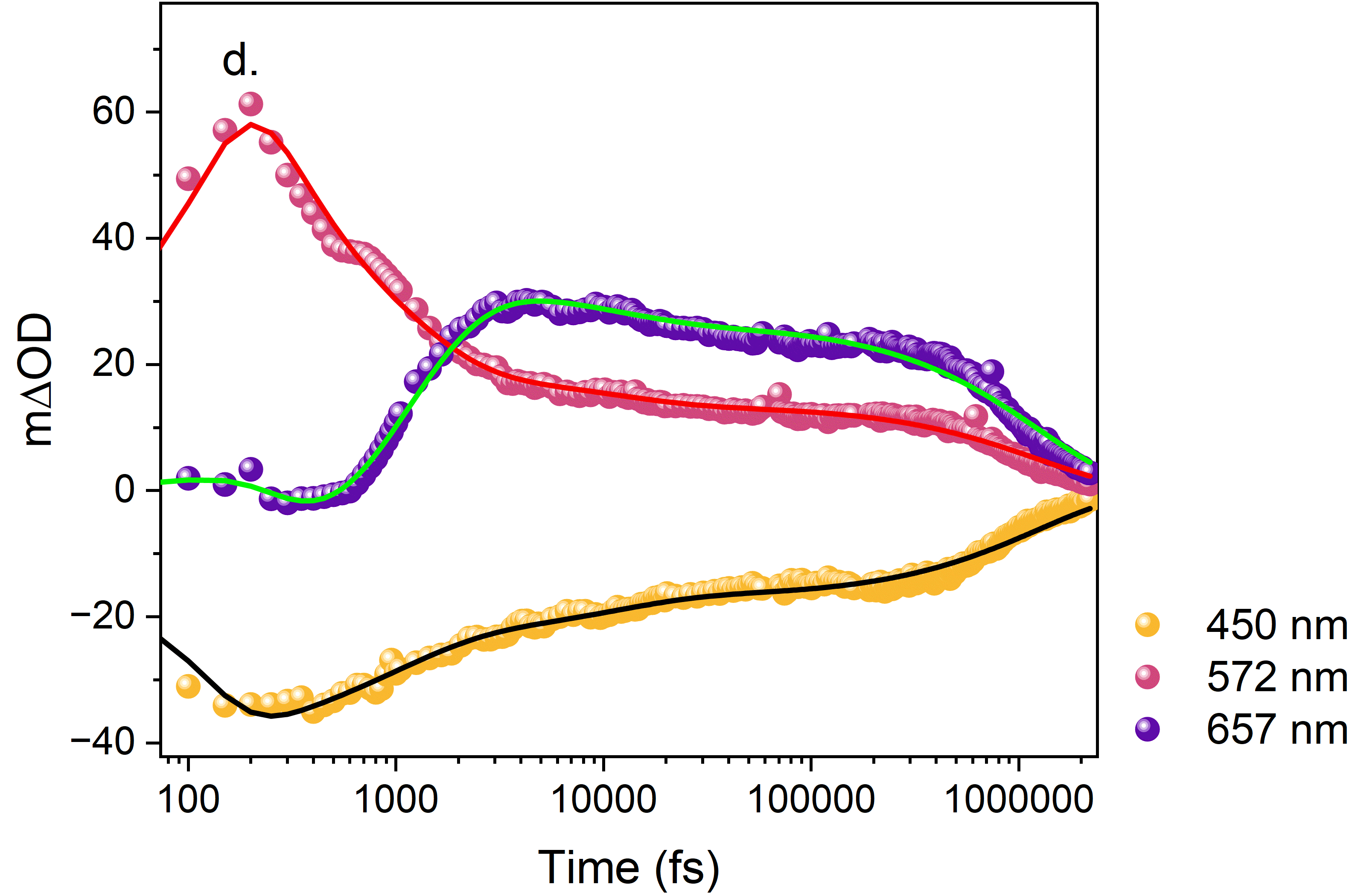


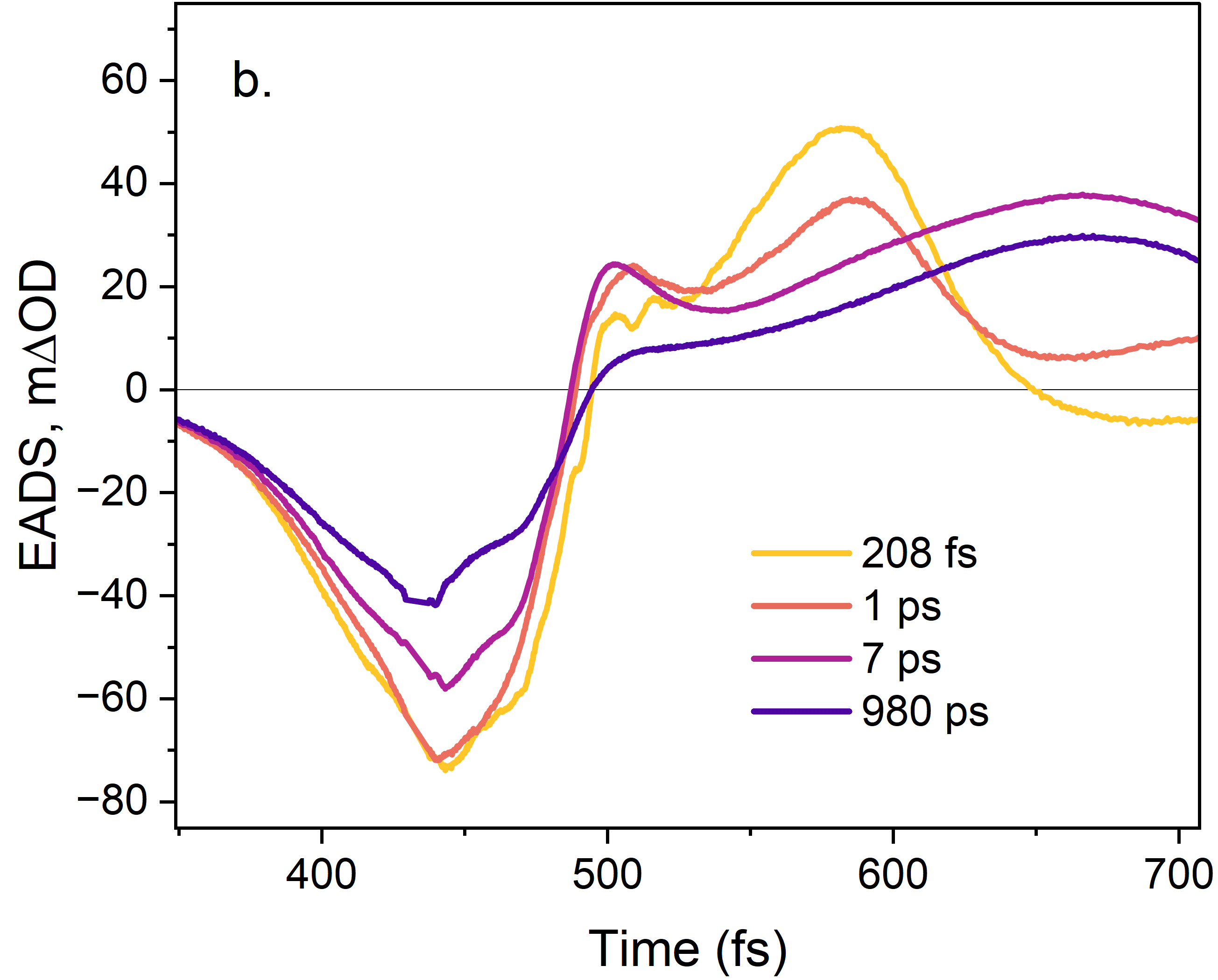

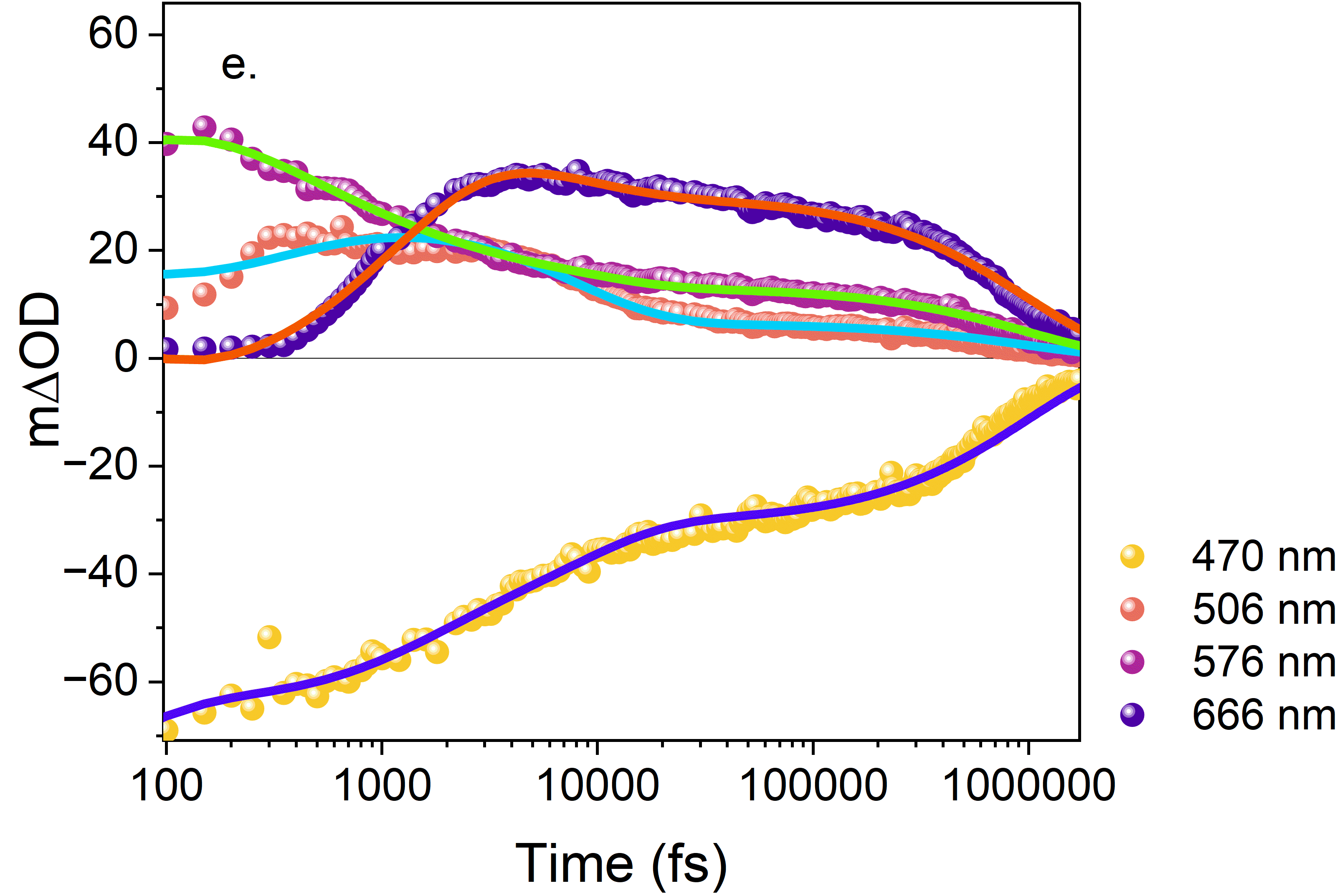


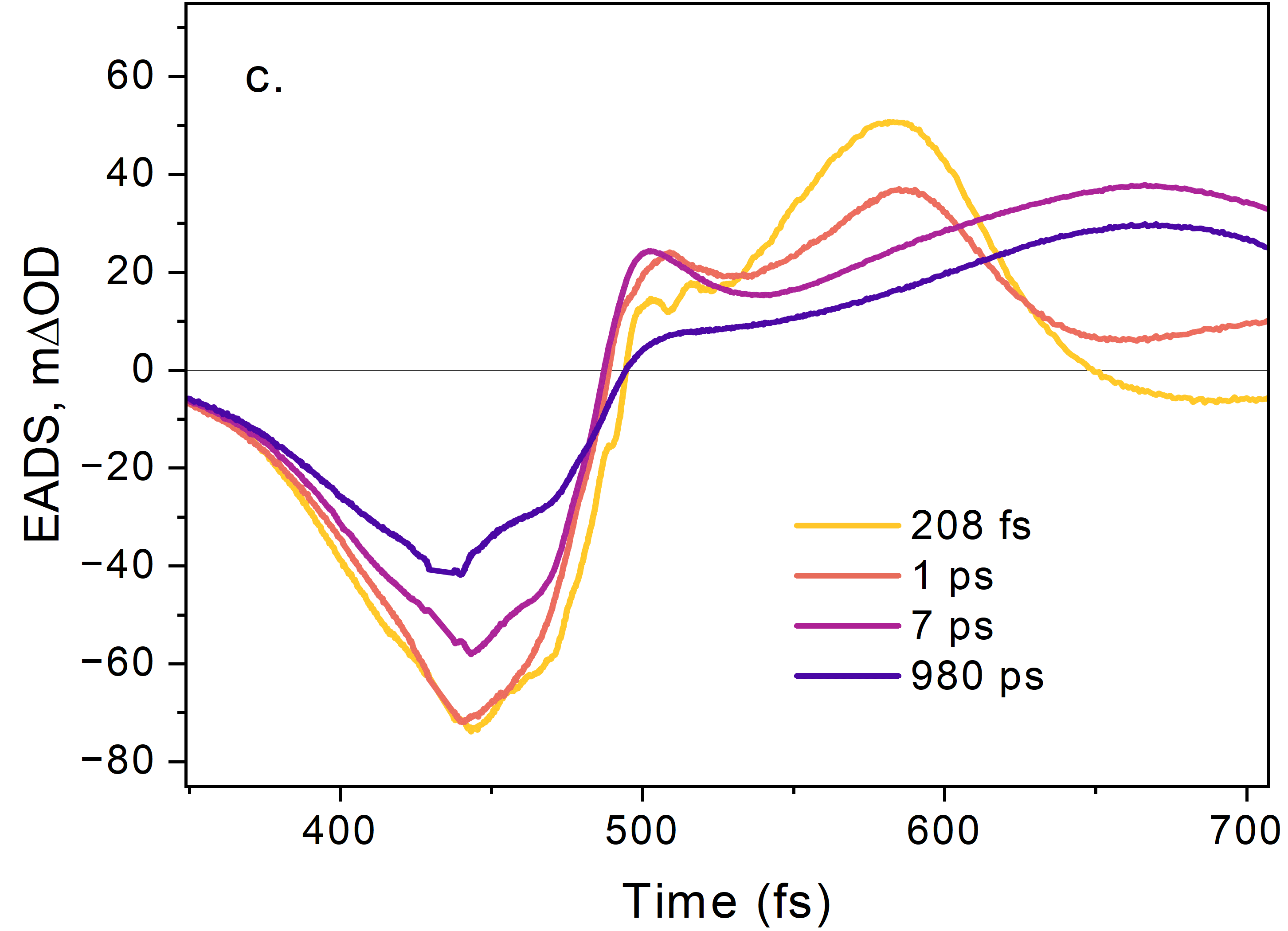

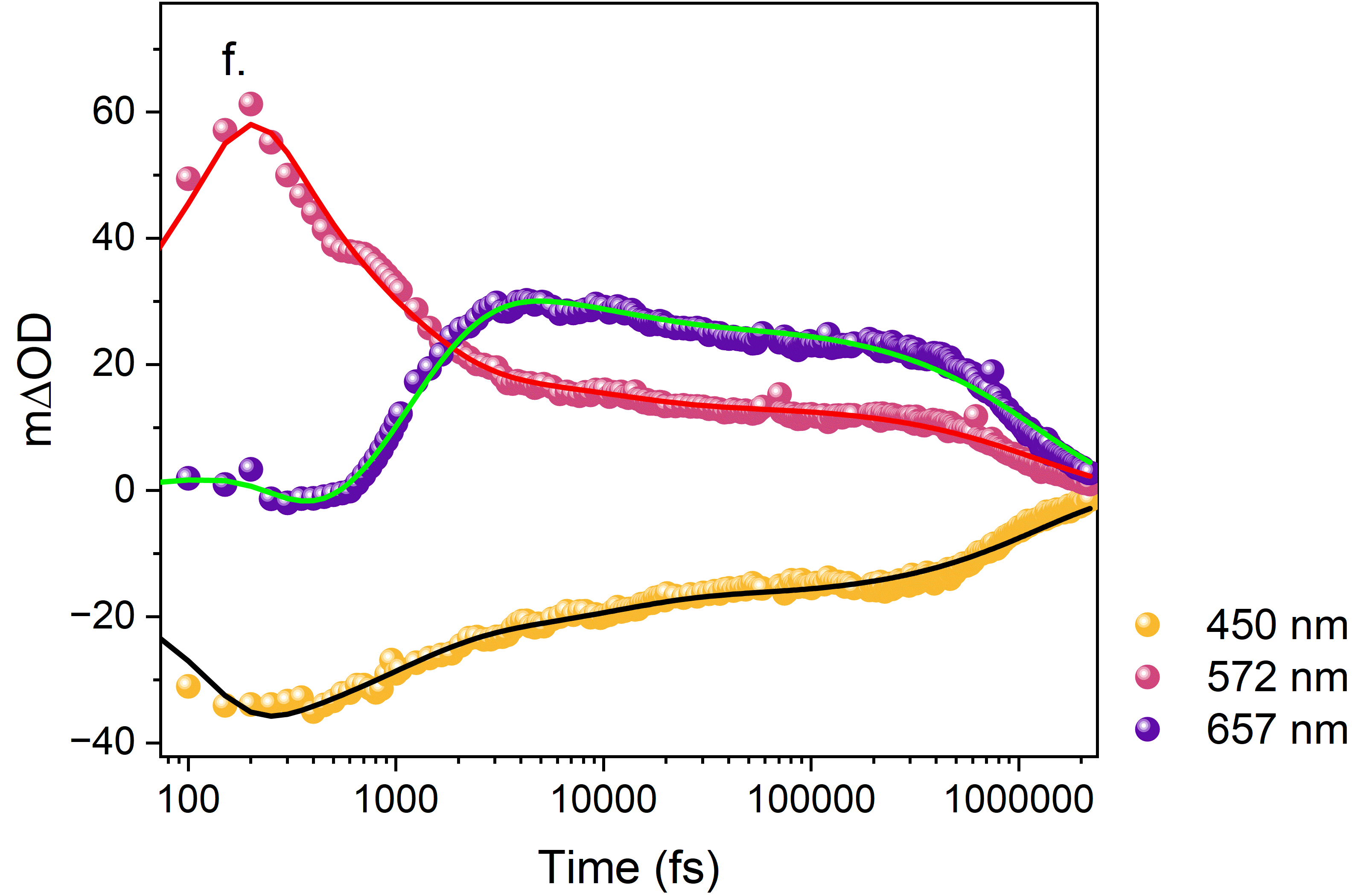


## Fig. S3 Evolution-associated difference spectra (EADS) derived from global fitting of the kinetic traces of the transient absorption data in (a) acetonitrile, (b) THF, and (c) ethylene glycol; (d), (e) and (f) show corresponding global exponential fit of kinetic traces presented at selected wavelengths in the respective solvents.

| Solvent | *ε* | *η (mPa s)* | τ_1_ (ps) | τ_2_ (ps) | τ_3_ (ps) | τ _4_ (ps) |
| --- | --- | --- | --- | --- | --- | --- |
| MeOH | 33 | 0.544 | 0.170 | 0.72 | 73 | 730 |
| ACN | 36.64 | 0.369 | 0.162 | 0.96 | 10 | 460 |
| THF | 7.52 | 0.456 | 0.280 | 1.00 | 7 | 980 |
| EG | 41.4 | 16.06 | -- | 1.5 | 96 | 1200 |

## Table S2. Comparison of the lifetimes of I, obtained from transient absorption spectroscopy, across different solvents as a function of their dielectric constant (*ε*) and viscosity (*η*).


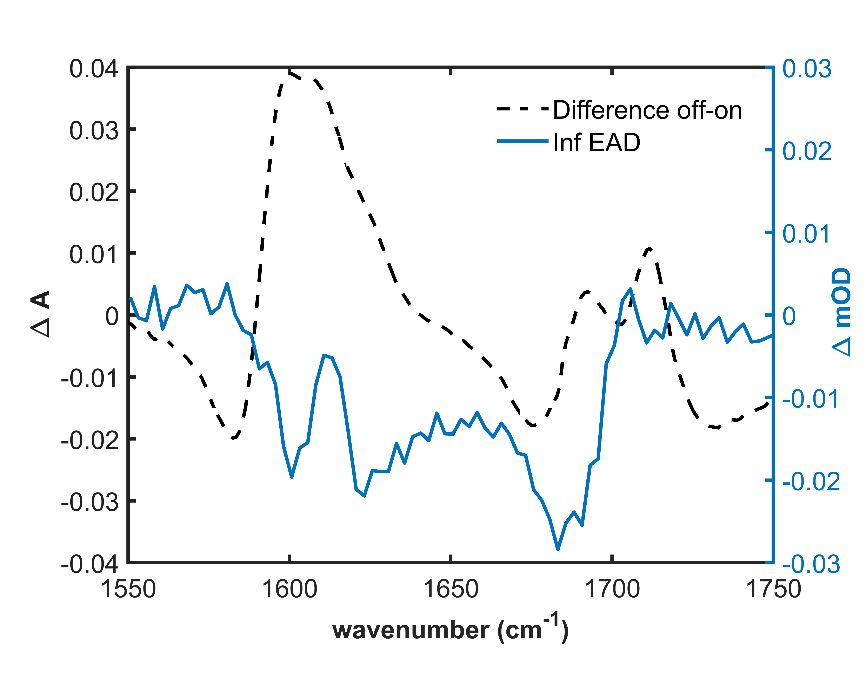


## Fig. S4. The Final EADS from the data analysis (Figure 3) is compared with the light minus dark difference spectrum recorded in FTIT. The same bands are observed but there is clearly some additional relaxation between 3 ns and the final spectrum.


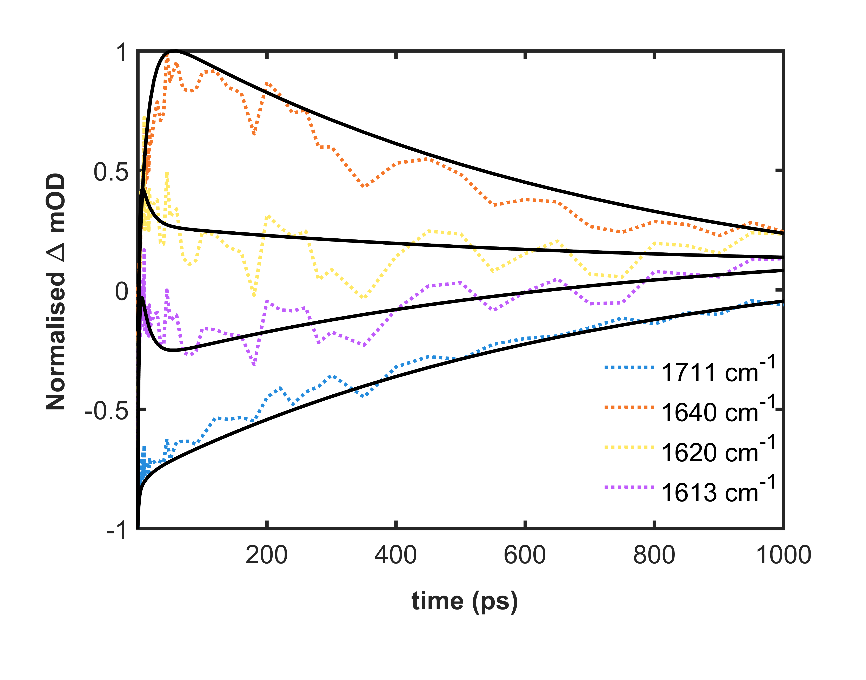


## Fig. S5. Time resolved IR data at key wavenumbers are compared with the fit (black lines) recovered form the global analysis.


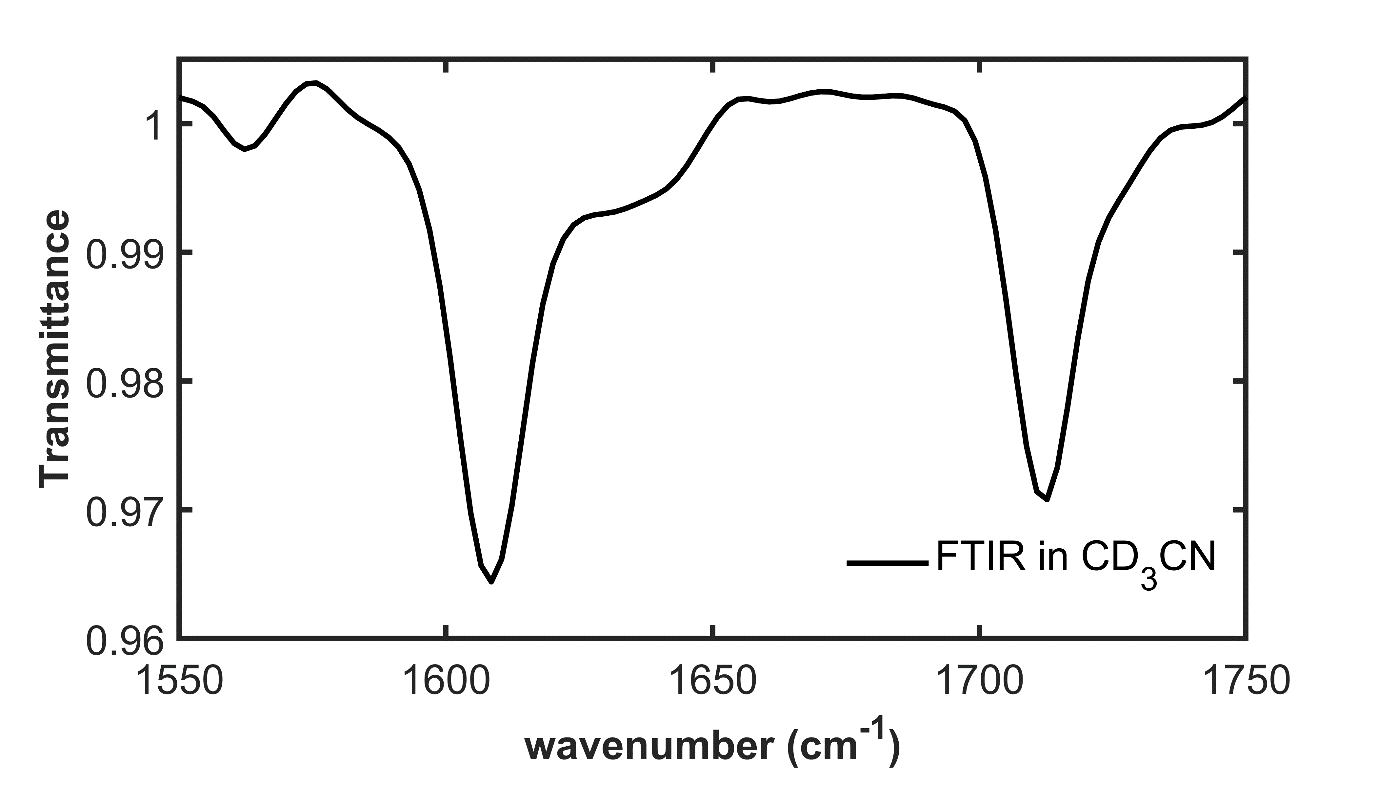


## Fig. S6 Experimental FTIR recorded in deuterated MeCN


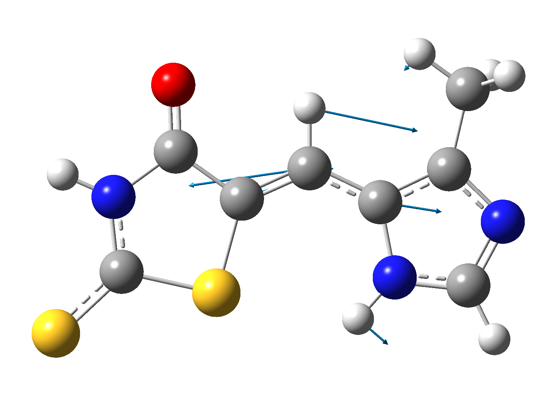

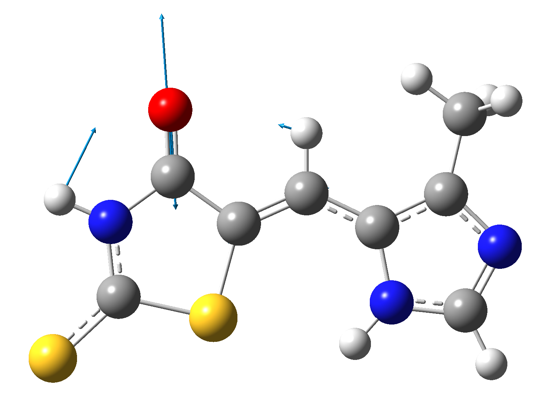


## Fig. S7 Displacement vectors illustrating normal modes associated with C=C stretch (calculated at 1711 cm^-1^) and C=O stretch (1866 cm^-1^) for S_0_-min (Z).


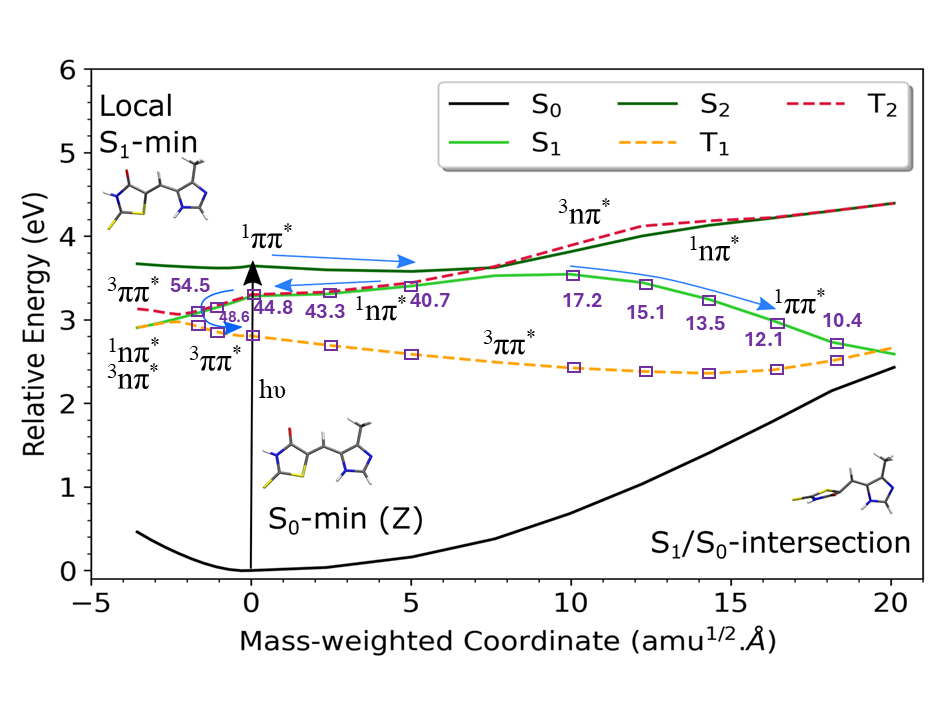


Fig. S8 Relative energies (with respect to ground state energy at S0-min (Z)) along linearly interpolated pathway from S_0_-min (Z) to S_1_/S_0_-intersection region and to the local S_1_-min. This illustrates that after internal conversion to S_1_ from S_2_, the population on S_1_ can evolve towards the S_1_/S_0_-intersection (high structural change) or towards the local S_1_-min (low structural change). However, the S_1_ state is a dark ^1^nπ* state only along the pathway towards the local S_1_-min whilst it is of ^1^ππ* character towards the S_1_/S_0_-intersection, which suggests a population transfer via intersystem crossing between the dark ^1^nπ* state and the triplet S_1_ state (^3^ππ*) is possible. SOCME (cm^-1^) along the LIIC (Table S3 and S4) also suggests that intersystem crossing via the dark ^1^nπ* state and the triplet S_1_ state (^3^nπ*) is more probable. The tables illustrate that after S_2_/S_1_ internal conversion (near mass-weighted coordinate ~7.6 Å), the maximum of S_1_-T_1_ SOCME increases along the mass-weighted coordinate up to an approximate T_1_/T_2_ crossing (at ~-2.3 Å), whereas the same decreases along the mass-weighted coordinate towards the S_2_/S_1_-intersection (near ~ 20.1 Å). Some of the max. SOCME between S_1_ and T_1_ is shown in cm^-1^ with purple-coloured boldfaced numbers.


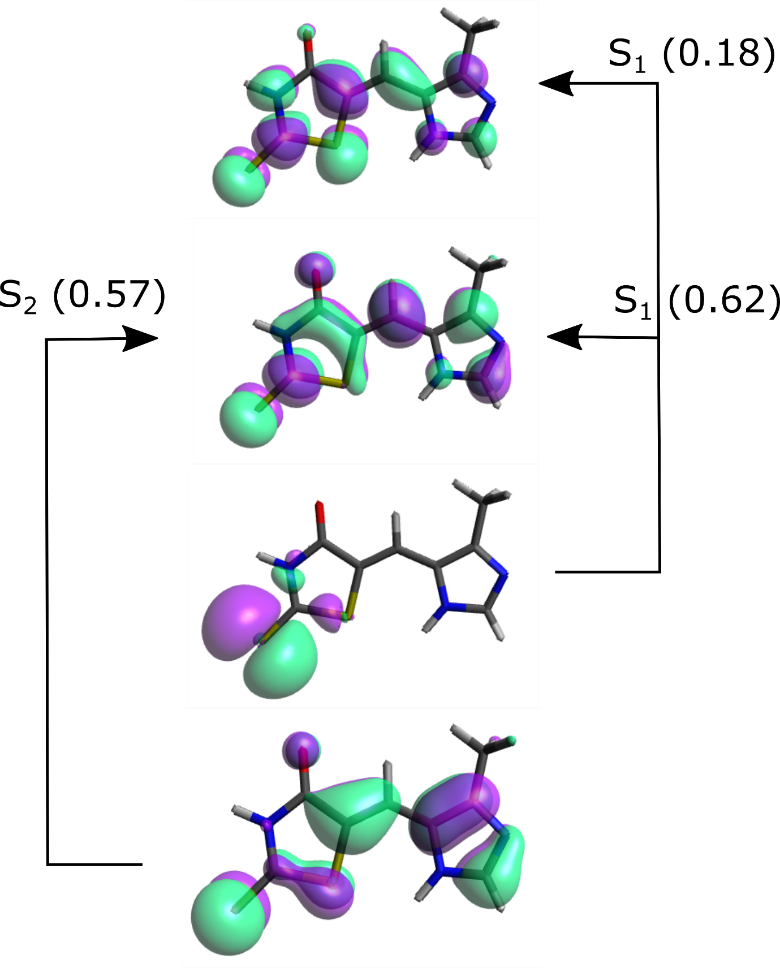


Fig. S9: Dominant orbital transitions along with their weights (in parenthesis) associated with S_1_ (nπ*) and S_2_ (ππ*) states at the S_0_-min (Z) based on CAS(14,12). This also illustrates that the n orbital from which excitation occurs in S_1,_ is the lone pair on the S atom of the C=S bond.


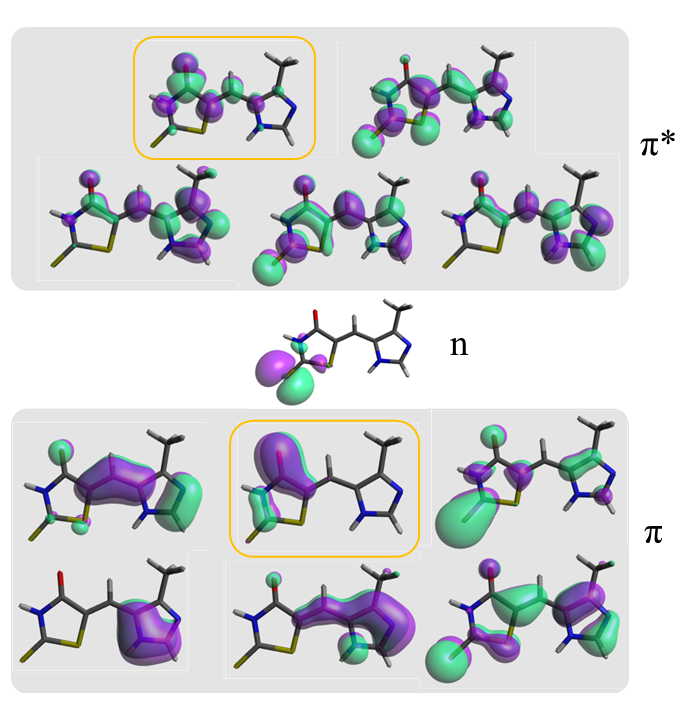


Fig S10: Active space orbitals used in the XMS-CASPT2/CAS(14,12)/cc-pVDZ single point calculations. For conical intersection optimization at the CASSCF(12,10)/cc-pVDZ level, the *π* and *π** orbitals associated with the C=O bond (enclosed by yellow boxes) were excluded. Isovalue: 0.03 a.u.


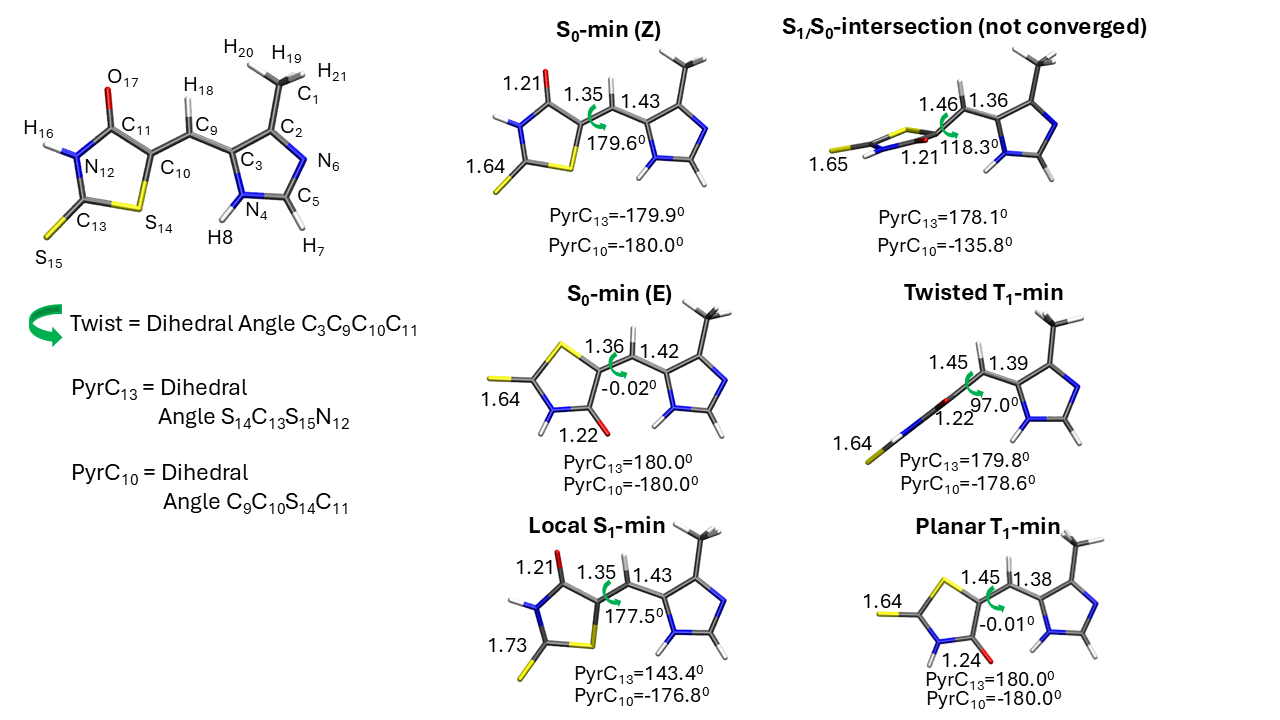


## Fig S11: Important critical points along key geometrical parameters and atom-indexing used. Pyramidalization at C_13_ is required to reach the local S_1_-min, whereas pyramidalization at C_10_ is required alongside twist to reach the vicinity of S_1_/S_0_-intersection. There is also a reversal of the bridging C-C bonds from singlet to triplet minima. Only four (S_0_-min (Z), local S_1_-min, twisted T_1_-min, S_1_/S_0_-intersection) out of the six located critical points have been used to explain the possible pathways after photoexcitation.


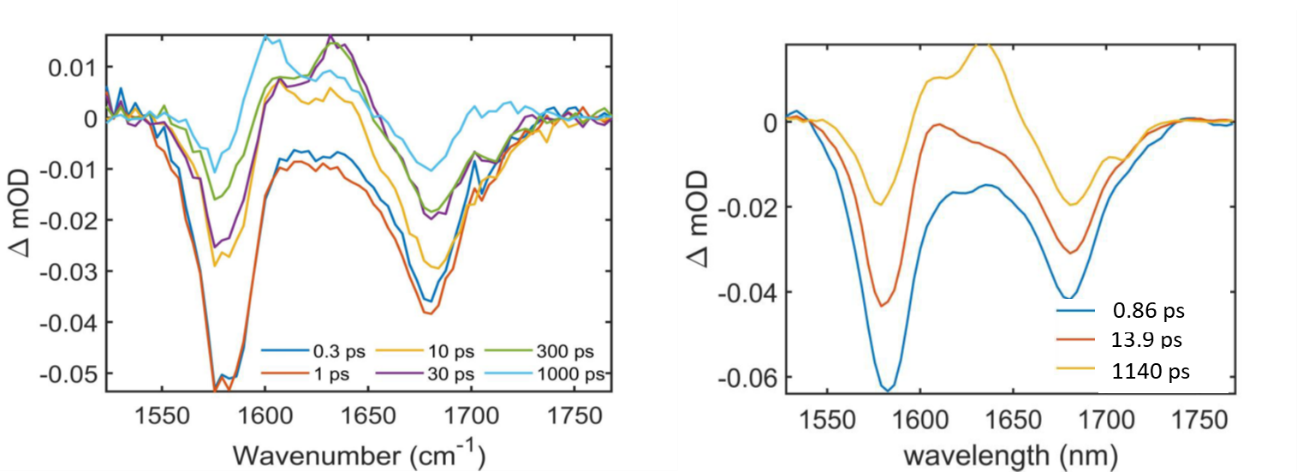


## Figure S12. Transient IR data for and E + Z photostationary state formed in CD_3_OD after irradiation at 365 nm. The pump wavelength was 350 nm. The data are strikingly similar to those in Figure 3 (Z state) suggesting similar kinetics for the E excited state relaxation.


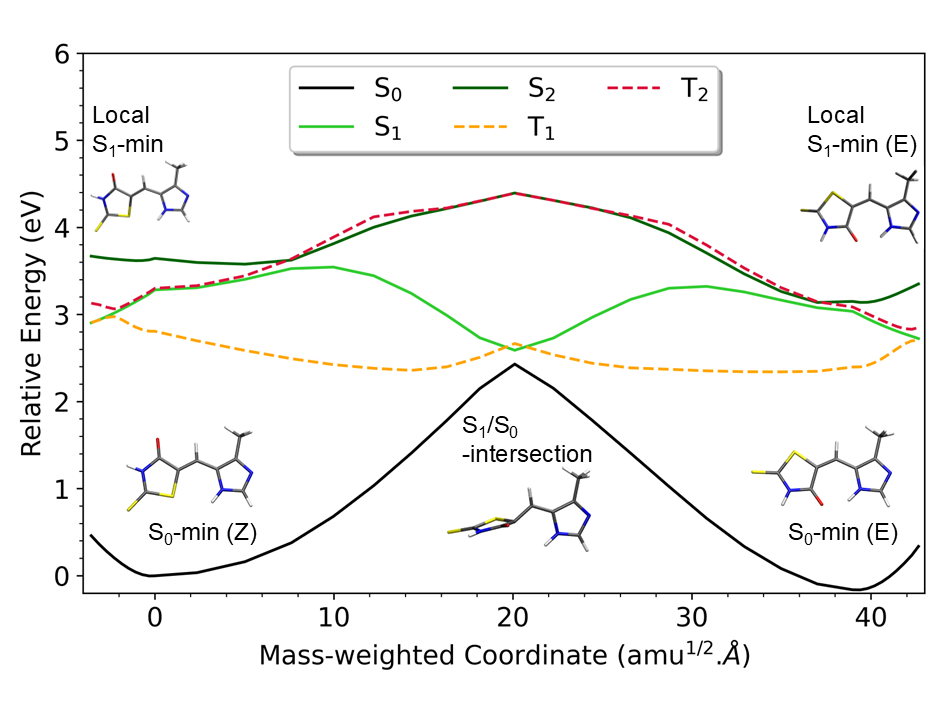


## Figure S13. The LIIC calculations from Fig 4 and S8 have been extended at the same level of theory (XMS-CASPT2/SA(5,3)-CAS(14,12)/cc-pVDZ) to include the E isomer in order to illustrate a comprehensive picture of the photoswitching mechanism. According to this LIIC, the photoswitching mechanism is expected to be very similar starting from either Z or E isomer. However, the S_2_ state is closer to the S_1_ state at the S_0_-min (E) compared to the S_0_-min (Z), which is expected to give a faster S_2_/S_1_ decay for the E isomer. There is also a barrier in the pathway to the S_1_/S_0_-intersection from the crossing region between S_2_ and S_1_ in the E isomer, which is expected to make it easier for population on S_1_ to move towards the local S_1_-min (E).

Table S3: SOCME (cm^-1^) for important singlet-triplet transitions along the LIIC between S_0_-min (Z) and local S_1_-min. Maximum of the S_1_-T_1_ SOCME is highlighted.

| Mass-weighted Coordinate (amu^1/2^.Å) | Transition | Magnetic quantum number for Triplet state | Absolute SOCME (cm^-1^) | Max. of SOCME (cm^-1^) |
| --- | --- | --- | --- | --- |
| 0 | S_1_ -T_1_ | +1 | 44.82 | 44.82 |
|  |  | 0 | 0.84 |  |
|  |  | -1 | 44.82 |  |
|  | S_1_ -T_2_ | +1 | 0.67 | 0.67 |
|  |  | 0 | 0.11 |  |
|  |  | -1 | 0.67 |  |
|  | S_2_ -T_1_ | +1 | 0.90 | 0.90 |
|  |  | 0 | 0.02 |  |
|  |  | -1 | 0.90 |  |
|  | S_2_ -T_2_ | +1 | 36.15 | 36.15 |
|  |  | 0 | 0.73 |  |
|  |  | -1 | 36.15 |  |
| -0.313 | S_1_ -T_1_ | +1 | 45.82 | 45.82 |
|  |  | 0 | 3.28 |  |
|  |  | -1 | 45.82 |  |
|  | S_1_ -T_2_ | +1 | 3.24 | 3.24 |
|  |  | 0 | 0.404 |  |
|  |  | -1 | 3.24 |  |
|  | S_2_ -T_1_ | +1 | 3.22 | 3.22 |
|  |  | 0 | 0.25 |  |
|  |  | -1 | 3.22 |  |
|  | S_2_ -T_2_ | +1 | 37.48 | 37.48 |
|  |  | 0 | 3.58 |  |
|  |  | -1 | 37.48 |  |
| -0.690 | S_1_ -T_1_ | +1 | 46.91 | 46.91 |
|  |  | 0 | 6.01 |  |
|  |  | -1 | 46.91 |  |
|  | S_1_ -T_2_ | +1 | 6.26 | 6.26 |
|  |  | 0 | 1.14 |  |
|  |  | -1 | 6.26 |  |
|  | S_2_ -T_1_ | +1 | 5.98 | 5.98 |
|  |  | 0 | 0.86 |  |
|  |  | -1 | 5.98 |  |
|  | S_2_ -T_2_ | +1 | 38.79 | 38.79 |
|  |  | 0 | 6.94 |  |
|  |  | -1 | 38.79 |  |
| -1.075 | S_1_ -T_1_ | +1 | 48.61 | 48.61 |
|  |  | 0 | 9.04 |  |
|  |  | -1 | 48.61 |  |
|  | S_1_ -T_2_ | +1 | 9.27 | 9.27 |
|  |  | 0 | 2.30 |  |
|  |  | -1 | 9.27 |  |
|  | S_2_ -T_1_ | +1 | 8.87 | 8.87 |
|  |  | 0 | 1.91 |  |
|  |  | -1 | 8.87 |  |
|  | S_2_ -T_2_ | +1 | 40.11 | 40.11 |
|  |  | 0 | 10.78 |  |
|  |  | -1 | 40.11 |  |
| -1.466 | S_1_ -T_1_ | +1 | 51.30 | 51.30 |
|  |  | 0 | 12.61 |  |
|  |  | -1 | 51.30 |  |
|  | S_1_ -T_2_ | +1 | 13.42 | 13.42 |
|  |  | 0 | 4.20 |  |
|  |  | -1 | 13.42 |  |
|  | S_2_ -T_1_ | +1 | 12.74 | 12.74 |
|  |  | 0 | 3.81 |  |
|  |  | -1 | 12.74 |  |
|  | S_2_ -T_2_ | +1 | 41.20 | 41.20 |
|  |  | 0 | 15.16 |  |
|  |  | -1 | 41.20 |  |
| -1.864 | S_1_ -T_1_ | +1 | 54.54 | 54.54 |
|  |  | 0 | 16.71 |  |
|  |  | -1 | 54.54 |  |
|  | S_1_ -T_2_ | +1 | 22.53 | 22.53 |
|  |  | 0 | 8.31 |  |
|  |  | -1 | 22.53 |  |
|  | S_2_ -T_1_ | +1 | 20.06 | 20.06 |
|  |  | 0 | 8.13 |  |
|  |  | -1 | 20.06 |  |
|  | S_2_ -T_2_ | +1 | 40.79 | 40.79 |
|  |  | 0 | 19.72 |  |
|  |  | -1 | 40.79 |  |
| -2.271 | S_1_ -T_1_ | +1 | 48.81 | 48.81 |
|  |  | 0 | 17.43 |  |
|  |  | -1 | 48.81 |  |
|  | S_1_ -T_2_ | +1 | 48.11 | 48.11 |
|  |  | 0 | 19.85 |  |
|  |  | -1 | 48.11 |  |
|  | S_2_ -T_1_ | +1 | 36.91 | 36.91 |
|  |  | 0 | 20.30 |  |
|  |  | -1 | 36.91 |  |
|  | S_2_ -T_2_ | +1 | 30.79 | 30.79 |
|  |  | 0 | 19.84 |  |
|  |  | -1 | 30.79 |  |
| -2.685 | S_1_ -T_1_ | +1 | 28.11 | 28.11 |
|  |  | 0 | 10.26 |  |
|  |  | -1 | 28.11 |  |
|  | S_1_ -T_2_ | +1 | 76.17 | 76.17 |
|  |  | 0 | 35.68 |  |
|  |  | -1 | 76.17 |  |
|  | S_2_ -T_1_ | +1 | 49.71 | 49.71 |
|  |  | 0 | 35.48 |  |
|  |  | -1 | 49.71 |  |
|  | S_2_ -T_2_ | +1 | 11.02 | 11.04 |
|  |  | 0 | 11.04 |  |
|  |  | -1 | 11.02 |  |
| -3.111 | S_1_ -T_1_ | +1 | 19.19 | 19.19 |
|  |  | 0 | 6.42 |  |
|  |  | -1 | 19.19 |  |
|  | S_1_ -T_2_ | +1 | 90.66 | 90.66 |
|  |  | 0 | 48.80 |  |
|  |  | -1 | 90.66 |  |
|  | S_2_ -T_1_ | +1 | 53.07 | 53.07 |
|  |  | 0 | 47.18 |  |
|  |  | -1 | 53.07 |  |
|  | S_2_ -T_2_ | +1 | 6.55 | 6.55 |
|  |  | 0 | 5.55 |  |
|  |  | -1 | 6.55 |  |
| -3.553 | S_1_ -T_1_ | +1 | 16.85 | 16.85 |
|  |  | 0 | 5.19 |  |
|  |  | -1 | 16.85 |  |
|  | S_1_ -T_2_ | +1 | 97.33 | 97.33 |
|  |  | 0 | 59.67 |  |
|  |  | -1 | 97.33 |  |
|  | S_2_ -T_1_ | +1 | 53.78 | 58.10 |
|  |  | 0 | 58.10 |  |
|  |  | -1 | 53.78 |  |
|  | S_2_ -T_2_ | +1 | 11.00 | 11.00 |
|  |  | 0 | 2.65 |  |
|  |  | -1 | 11.00 |  |

Table S4: SOCME (cm^-1^) for important singlet-triplet transitions along the LIIC between S_0_-min (Z) and S_1_/S_0_-intersection. Maximum of the S_1_-T_1_ SOCME is highlighted.

| Mass-weighted Coordinate (amu^1/2^.Å) | Transition | Magnetic quantum number for Triplet state | Absolute SOCME (cm^-1^) | Max. of SOCME (cm^-1^) |
| --- | --- | --- | --- | --- |
| 0 | S_1_ -T_1_ | +1 | 44.82 | 44.82 |
|  |  | 0 | 0.84 |  |
|  |  | -1 | 44.82 |  |
|  | S_1_ -T_2_ | +1 | 0.67 | 0.67 |
|  |  | 0 | 0.11 |  |
|  |  | -1 | 0.67 |  |
|  | S_2_ -T_1_ | +1 | 0.90 | 0.90 |
|  |  | 0 | 0.02 |  |
|  |  | -1 | 0.90 |  |
|  | S_2_ -T_2_ | +1 | 36.15 | 36.15 |
|  |  | 0 | 0.73 |  |
|  |  | -1 | 36.15 |  |
| 2.357 | S_1_ -T_1_ | +1 | 43.28 | 43.28 |
|  |  | 0 | 3.94 |  |
|  |  | -1 | 43.28 |  |
|  | S_1_ -T_2_ | +1 | 2.26 | 2.26 |
|  |  | 0 | 0.11 |  |
|  |  | -1 | 2.26 |  |
|  | S_2_ -T_1_ | +1 | 2.38 | 2.38 |
|  |  | 0 | 0.15 |  |
|  |  | -1 | 2.38 |  |
|  | S_2_ -T_2_ | +1 | 35.23 | 35.23 |
|  |  | 0 | 2.59 |  |
|  |  | -1 | 35.23 |  |
| 5.033 | S_1_ -T_1_ | +1 | 40.74 | 40.74 |
|  |  | 0 | 8.69 |  |
|  |  | -1 | 40.74 |  |
|  | S_1_ -T_2_ | +1 | 6.14 | 6.14 |
|  |  | 0 | 1.15 |  |
|  |  | -1 | 6.14 |  |
|  | S_2_ -T_1_ | +1 | 6.32 | 6.32 |
|  |  | 0 | 1.19 |  |
|  |  | -1 | 6.32 |  |
|  | S_2_ -T_2_ | +1 | 33.43 | 33.43 |
|  |  | 0 | 5.91 |  |
|  |  | -1 | 33.43 |  |
| 7.624 | S_1_ -T_1_ | +1 | 29.86 | 29.86 |
|  |  | 0 | 9.86 |  |
|  |  | -1 | 29.86 |  |
|  | S_1_ -T_2_ | +1 | 23.24 | 23.24 |
|  |  | 0 | 6.70 |  |
|  |  | -1 | 23.24 |  |
|  | S_2_ -T_1_ | +1 | 28.23 | 28.23 |
|  |  | 0 | 9.49 |  |
|  |  | -1 | 28.23 |  |
|  | S_2_ -T_2_ | +1 | 22.29 | 22.29 |
|  |  | 0 | 5.91 |  |
|  |  | -1 | 22.29 |  |
| 9.968 | S_1_ -T_1_ | +1 | 17.15 | 17.15 |
|  |  | 0 | 6.32 |  |
|  |  | -1 | 17.15 |  |
|  | S_1_ -T_2_ | +1 | 29.02 | 29.02 |
|  |  | 0 | 10.93 |  |
|  |  | -1 | 29.02 |  |
|  | S_2_ -T_1_ | +1 | 40.69 | 40.69 |
|  |  | 0 | 18.50 |  |
|  |  | -1 | 40.69 |  |
|  | S_2_ -T_2_ | +1 | 10.00 | 10.00 |
|  |  | 0 | 2.71 |  |
|  |  | -1 | 10.00 |  |
| 12.220 | S_1_ -T_1_ | +1 | 14.95 | 14.95 |
|  |  | 0 | 4.98 |  |
|  |  | -1 | 14.95 |  |
|  | S_1_ -T_2_ | +1 | 31.89 | 31.89 |
|  |  | 0 | 14.72 |  |
|  |  | -1 | 31.89 |  |
|  | S_2_ -T_1_ | +1 | 48.24 | 48.24 |
|  |  | 0 | 27.20 |  |
|  |  | -1 | 48.24 |  |
|  | S_2_ -T_2_ | +1 | 6.68 | 6.68 |
|  |  | 0 | 1.18 |  |
|  |  | -1 | 6.68 |  |
| 14.322 | S_1_ -T_1_ | +1 | 13.49 | 13.49 |
|  |  | 0 | 3.32 |  |
|  |  | -1 | 13.49 |  |
|  | S_1_ -T_2_ | +1 | 37.73 | 37.73 |
|  |  | 0 | 19.45 |  |
|  |  | -1 | 37.73 |  |
|  | S_2_ -T_1_ | +1 | 54.06 | 54.06 |
|  |  | 0 | 35.15 |  |
|  |  | -1 | 54.06 |  |
|  | S_2_ -T_2_ | +1 | 4.34 | 4.34 |
|  |  | 0 | 1.00 |  |
|  |  | -1 | 4.34 |  |
| 16.300 | S_1_ -T_1_ | +1 | 12.07 | 12.07 |
|  |  | 0 | 1.78 |  |
|  |  | -1 | 12.07 |  |
|  | S_1_ -T_2_ | +1 | 39.07 | 39.07 |
|  |  | 0 | 21.93 |  |
|  |  | -1 | 39.07 |  |
|  | S_2_ -T_1_ | +1 | 56.53 | 56.52 |
|  |  | 0 | 40.24 |  |
|  |  | -1 | 56.52 |  |
|  | S_2_ -T_2_ | +1 | 3.11 | 3.11 |
|  |  | 0 | 1.49 |  |
|  |  | -1 | 3.11 |  |
| 18.150 | S_1_ -T_1_ | +1 | 10.40 | 10.40 |
|  |  | 0 | 0.44 |  |
|  |  | -1 | 10.40 |  |
|  | S_1_ -T_2_ | +1 | 41.35 | 41.35 |
|  |  | 0 | 25.74 |  |
|  |  | -1 | 41.35 |  |
|  | S_2_ -T_1_ | +1 | 56.78 | 56.78 |
|  |  | 0 | 42.42 |  |
|  |  | -1 | 56.78 |  |
|  | S_2_ -T_2_ | +1 | 2.23 | 2.23 |
|  |  | 0 | 1.11 |  |
|  |  | -1 | 2.23 |  |
| 20.100 | S_1_ -T_1_ | +1 | 0.35 | 0.35 |
|  |  | 0 | 0.14 |  |
|  |  | -1 | 0.35 |  |
|  | S_1_ -T_2_ | +1 | 57.69 | 57.69 |
|  |  | 0 | 41.82 |  |
|  |  | -1 | 57.69 |  |
|  | S_2_ -T_1_ | +1 | 56.34 | 56.34 |
|  |  | 0 | 42.96 |  |
|  |  | -1 | 56.34 |  |
|  | S_2_ -T_2_ | +1 | 1.70 | 1.70 |
|  |  | 0 | 0.72 |  |
|  |  | -1 | 1.70 |  |

**Optimized Critical Points at (TD)-DFT/ωB97x-D/6-31G(d,p) level:**

**S_0_-min (Z):**

C -4.090446 1.539551 0.106192

C -3.425593 0.205488 0.042495

C -2.067506 -0.088118 -0.003512

N -2.012605 -1.474278 -0.043072

C -3.285556 -1.923615 -0.022353

N -4.162068 -0.943632 0.030965

H -3.524713 -2.977314 -0.048825

H -1.188414 -2.050581 -0.099890

C -0.953849 0.804884 -0.007277

C 0.369758 0.550674 -0.019170

C 1.349192 1.657531 -0.030665

N 2.639100 1.115878 -0.047960

C 2.802515 -0.238208 -0.049561

S 1.208427 -1.003814 -0.022413

S 4.220815 -1.051450 -0.069533

H 3.448010 1.722274 -0.059279

O 1.119540 2.845532 -0.027802

H -1.199654 1.864411 0.000758

H -4.641159 1.642639 1.045429

H -3.376012 2.361954 0.035358

H -4.811886 1.636436 -0.709182

**S_0_-min (E):**

C 3.789652 -2.105078 -0.024311

C 3.400427 -0.664081 -0.007354

C 2.121475 -0.098730 -0.000588

N 2.338462 1.265720 -0.006008

C 3.665441 1.452744 -0.015876

N 4.342286 0.313545 -0.017130

H 4.111270 2.437182 -0.019647

H 1.573734 1.941809 -0.001798

C 0.862819 -0.760267 0.008646

C -0.409792 -0.282651 0.010803

C -0.869025 1.111435 0.004250

N -2.255611 1.134004 0.007060

C -2.956421 -0.043962 0.016389

S -1.814249 -1.374077 0.022194

S -4.587481 -0.187116 0.021570

H -2.748964 2.016833 0.003490

O -0.212214 2.145084 -0.001918

H 0.958626 -1.843967 0.013061

H 4.614556 -2.270461 0.672020

H 2.964399 -2.763846 0.255369

H 4.136391 -2.397001 -1.020442

**Local S_1_-min:**

C 0.002914 -0.005463 0.034746

C 0.690939 -1.327744 -0.033134

C 2.052863 -1.593677 -0.065195

N 2.137715 -2.978049 -0.093939

C 0.872136 -3.455190 -0.093026

N -0.023051 -2.493714 -0.046861

H 0.655593 -4.513448 -0.128641

H 2.975033 -3.525417 -0.216153

C 3.160955 -0.688767 -0.066938

C 4.477263 -0.939970 0.077599

C 5.476804 0.151498 -0.010869

N 6.745507 -0.388600 0.134992

C 6.872512 -1.744720 0.373824

S 5.267442 -2.471329 0.464810

S 8.198478 -2.665001 -0.255006

H 7.543804 0.228454 0.105426

O 5.253048 1.333228 -0.180886

H 2.923973 0.363657 -0.205761

H -0.455639 0.138613 1.017774

H 0.690457 0.824103 -0.143447

H -0.795699 0.037648 -0.709795

**Twisted T_1_-min:**

C 0.002914 -0.005463 0.034746

C 0.690939 -1.327744 -0.033134

C 2.052863 -1.593677 -0.065195

N 2.137715 -2.978049 -0.093939

C 0.872136 -3.455190 -0.093026

N -0.023051 -2.493714 -0.046861

H 0.655593 -4.513448 -0.128641

H 2.975033 -3.525417 -0.216153

C 3.160955 -0.688767 -0.066938

C 4.477263 -0.939970 0.077599

C 5.476804 0.151498 -0.010869

N 6.745507 -0.388600 0.134992

C 6.872512 -1.744720 0.373824

S 5.267442 -2.471329 0.464810

S 8.198478 -2.665001 -0.255006

H 7.543804 0.228454 0.105426

O 5.253048 1.333228 -0.180886

H 2.923973 0.363657 -0.205761

H -0.455639 0.138613 1.017774

H 0.690457 0.824103 -0.143447

H -0.795699 0.037648 -0.709795

**Planar T_1_-min:**

C 3.818200 -2.086085 -0.003329

C 3.450134 -0.646906 0.016501

C 2.105335 -0.107853 0.008716

N 2.302422 1.251348 0.034230

C 3.644332 1.473287 0.054973

N 4.353188 0.352558 0.044871

H 4.061654 2.469591 0.077143

H 1.521161 1.913794 0.035984

C 0.913750 -0.804756 -0.018140

C -0.426726 -0.258481 -0.025612

C -0.883098 1.108164 -0.007718

N -2.285904 1.126297 -0.025163

C -2.967900 -0.042110 -0.053946

S -1.767995 -1.341811 -0.061308

S -4.594996 -0.269438 -0.078403

H -2.776627 2.010210 -0.015988

O -0.235092 2.164448 0.018786

H 0.994535 -1.885324 -0.035509

H 4.903803 -2.185975 0.008966

H 3.405632 -2.612826 0.865130

H 3.428410 -2.582688 -0.899623

**Local S_1_-min (E):**

               C                   3.781239   -2.106613   -0.002187

                C                   3.392678   -0.664841    0.021904

                C                   2.115995   -0.100951   -0.006670

                N                   2.330584    1.262164    0.011728

                C                   3.658974    1.452053    0.047935

                N                   4.335882    0.314783    0.055305

                H                   4.102005    2.437458    0.071375

                H                   1.561878    1.935011    0.000470

                C                   0.852873   -0.762953   -0.046093

               C                  -0.416061   -0.277264   -0.081707

               C                  -0.877190    1.122435   -0.064447

               N                  -2.252789    1.150523   -0.082837

               C                  -2.953103   -0.038570   -0.166924

               S                  -1.812219   -1.369417   -0.221068

               S                  -4.503279   -0.245804    0.581328

               H                  -2.713626    2.049064   -0.072282

               O                  -0.210629    2.156072   -0.041345

               H                   0.946778   -1.846485   -0.055620

               H                   4.632670   -2.267350    0.662432

               H                   2.966568   -2.761300    0.317101

               H                   4.086553   -2.410896   -1.008618

**S_1_/S_0_-intersection at SA5-CASSCF(12,10)/cc-pVDZ level (not converged):**

C 1.347803904507e-01 2.810260140499e-02 -2.703474929985e-01

C -5.881967958454e-01 -1.230994856811e+00 8.317109238013e-02

C -2.021585764658e+00 -1.456188480356e+00 6.539093385917e-02

N -2.142428959957e+00 -2.776117253598e+00 4.860981621196e-01

C -9.527121806505e-01 -3.242893108211e+00 7.167886921551e-01

N 2.971884288909e-02 -2.319521915154e+00 4.731354457771e-01

H -7.589347680008e-01 -4.242864575395e+00 1.065444170369e+00

H -3.059120024886e+00 -3.187675487596e+00 5.916983882005e-01

C -3.129861621172e+00 -7.183276166266e-01 -2.212074890714e-01

C -4.454872364405e+00 -1.322135127757e+00 -1.370054525510e-01

C -5.364011149177e+00 -7.664258582032e-01 7.986978932919e-01

N -6.680026362172e+00 -8.971035897033e-01 3.234643152656e-01

C -6.878982548107e+00 -1.267969367130e+00 -9.605280250964e-01

S -5.322893623558e+00 -1.610667616349e+00 -1.693946380983e+00

S -8.328962799478e+00 -1.424233295151e+00 -1.723876266891e+00

H -7.448997741092e+00 -6.070420131357e-01 8.927782606604e-01

O -5.135209670701e+00 -2.406673791249e-01 1.864489429393e+00

H -2.960804008301e+00 3.217349761944e-01 -5.040157095424e-01

H 9.132780812671e-01 -1.896220618666e-01 -1.001696553611e+00

H -5.404463892445e-01 7.771021119177e-01 -6.789633633711e-01

H 6.133125405314e-01 4.352108582098e-01 6.212553641894e-01

[1] M. Kondo, I. A. Heisler, J. Conyard, J. P. H. Rivett, S. R. Meech, *J. Phys. Chem. B* **2009**, *113*, 1632–1639.

[2] P. Roy, G. Bressan, J. Gretton, A. N. Cammidge, S. R. Meech, *Angew. Chemie - Int. Ed.* **2021**, *60*, 10568–10572.

[3] W. Kohn, A. D. Becke, R. G. Parr, *J. Phys. Chem.* **1996**, *100*, 12974–12980.

[4] T. Ziegler, *Chem. Rev.* **1991**, *91*, 651–667.

[5] J.-D. Chai, M. Head-Gordon, *Phys. Chem. Chem. Phys.* **2008**, *10*, 6615.

[6] R. Ditchfield, W. J. Hehre, J. A. Pople, *J. Chem. Phys.* **1971**, *54*, 724–728.

[7] E. Runge, E. K. U. Gross, *Phys. Rev. Lett.* **1984**, *52*, 997–1000.

[8] R. Bauernschmitt, R. Ahlrichs, *Chem. Phys. Lett.* **1996**, *256*, 454–464.

[9] F. Furche, R. Ahlrichs, *J. Chem. Phys.* **2002**, *117*, 7433–7447.

[10] M. J. Frisch, G. W. Trucks, H. B. Schlegel, G. E. Scuseria, M. A. Robb, J. R. Cheeseman, G. Scalmani, V. Barone, B. Mennucci, G. A. Petersson, H. Nakatsuji, M. Caricato, X. Li, H. P. Hratchian, A. F. Izmaylov, J. Bloino, G. Zheng, J. L. Sonnenberg, M. Hada, M. Ehara, K. Toyota, R. Fukuda, J. Hasegawa, M. Ishida, T. Nakajima, Y. Honda, O. Kitao, H. Nakai, T. Vreven, J. A. Montgomery, J. E. Peralta, F. Ogliaro, M. Bearpark, J. J. Heyd, E. Brothers, K. N. Kudin, V. N. Staroverov, R. Kobayashi, J. Normand, K. Raghavachari, A. Rendell, J. C. Burant, S. S. Iyengar, J. Tomasi, M. Cossi, N. Rega, J. M. Millam, M. Klene, J. E. Knox, J. B. Cross, V. Bakken, C. Adamo, J. Jaramillo, R. Gomperts, R. E. Stratmann, O. Yazyev, A. J. Austin, R. Cammi, C. Pomelli, J. W. Ochterski, R. L. Martin, K. Morokuma, V. G. Zakrzewski, G. A. Voth, P. Salvador, J. J. Dannenberg, S. Dapprich, A. D. Daniels, O. Farkas, J. B. Foresman, J. V Ortiz, J. Cioslowski, D. J. Fox, *Gaussian 16, Revis. B.01, Gaussian, Inc., Wallingford CT* **2016**.

[11] B. O. Roos, P. R. Taylor, P. E. M. Sigbahn, *Chem. Phys.* **1980**, *48*, 157–173.

[12] T. H. Dunning, *J. Chem. Phys.* **1989**, *90*, 1007–1023.

[13] **N.d.**

[14] T. Shiozaki, *WIREs Comput Mol Sci.* **2018**, *8*, e1331.

[15] J. Finley, P.-Å. Malmqvist, B. O. Roos, L. Serrano-Andrés, *Chem. Phys. Lett.* **1998**, *288*, 299–306.

[16] A. A. Granovsky, *J. Chem. Phys.* **2011**, *134*, 214113.

[17] T. Shiozaki, W. Gy\Horffy, P. Celani, H.-J. Werner, **2011**.

[18] I. Fdez. Galván, M. Vacher, A. Alavi, C. Angeli, F. Aquilante, J. Autschbach, J. J. Bao, S. I. Bokarev, N. A. Bogdanov, R. K. Carlson, L. F. Chibotaru, J. Creutzberg, N. Dattani, M. G. Delcey, S. S. Dong, A. Dreuw, L. Freitag, L. M. Frutos, L. Gagliardi, F. Gendron, A. Giussani, L. González, G. Grell, M. Guo, C. E. Hoyer, M. Johansson, S. Keller, S. Knecht, G. Kovačević, E. Källman, G. Li Manni, M. Lundberg, Y. Ma, S. Mai, J. P. Malhado, P. Å. Malmqvist, P. Marquetand, S. A. Mewes, J. Norell, M. Olivucci, M. Oppel, Q. M. Phung, K. Pierloot, F. Plasser, M. Reiher, A. M. Sand, I. Schapiro, P. Sharma, C. J. Stein, L. K. Sørensen, D. G. Truhlar, M. Ugandi, L. Ungur, A. Valentini, S. Vancoillie, V. Veryazov, O. Weser, T. A. Wesołowski, P.-O. Widmark, S. Wouters, A. Zech, J. P. Zobel, R. Lindh, *J. Chem. Theory Comput.* **2019**, *15*, 5925–5964.

[19] F. Aquilante, J. Autschbach, A. Baiardi, S. Battaglia, V. A. Borin, L. F. Chibotaru, I. Conti, L. De Vico, M. Delcey, I. Fdez. Galván, N. Ferré, L. Freitag, M. Garavelli, X. Gong, S. Knecht, E. D. Larsson, R. Lindh, M. Lundberg, P. Å. Malmqvist, A. Nenov, J. Norell, M. Odelius, M. Olivucci, T. B. Pedersen, L. Pedraza-González, Q. M. Phung, K. Pierloot, M. Reiher, I. Schapiro, J. Segarra-Martí, F. Segatta, L. Seijo, S. Sen, D.-C. Sergentu, C. J. Stein, L. Ungur, M. Vacher, A. Valentini, V. Veryazov, *J. Chem. Phys.* **2020**, *152*, 214117.
